# Interfacial Charge Transfer Complexes in TiO_2_-Enediol Hybrids Synthesized by Sol–Gel

**DOI:** 10.1021/acs.langmuir.1c02939

**Published:** 2022-01-28

**Authors:** Claudio Imparato, Gerardino D’Errico, Wojciech Macyk, Marcin Kobielusz, Giuseppe Vitiello, Antonio Aronne

**Affiliations:** † Department of Chemical, Materials and Production Engineering, University of Naples Federico II, P.le V. Tecchio 80, 80125 Napoli, Italy; ‡ Department of Chemical Sciences, University of Naples Federico II, Via Cinthia, 80126 Napoli, Italy; § Faculty of Chemistry, Jagiellonian University, ul. Gronostajowa 2, Kraków 30-387, Poland

## Abstract

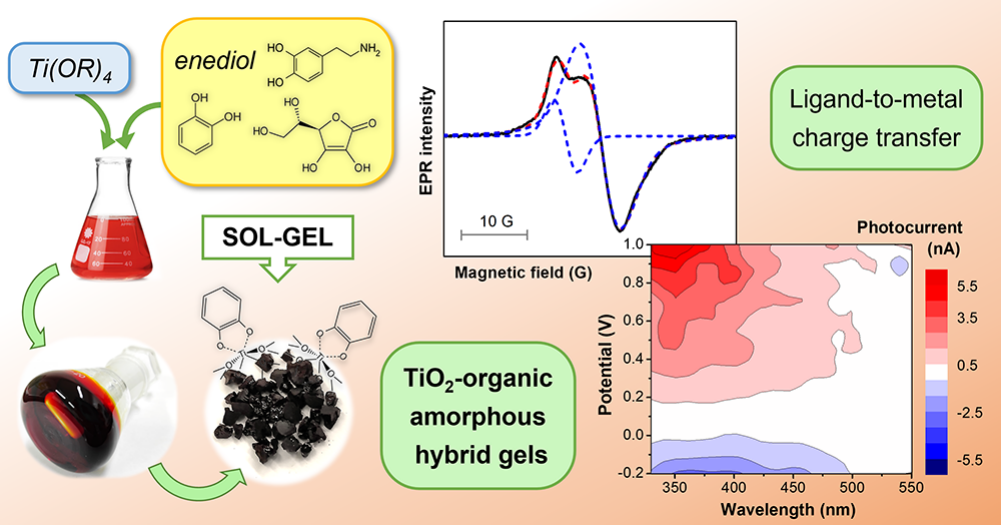

Metal oxide-organic hybrid semiconductors exhibit specific properties depending not only on their composition but also on the synthesis procedure, and particularly on the functionalization method, determining the interaction between the two components. Surface adsorption is the most common way to prepare organic-modified metal oxides. Here a simple sol–gel route is described as an alternative, finely controlled strategy to synthesize titanium oxide-based materials containing organic molecules coordinated to the metal. The effect of the molecular structure of the ligands on the surface properties of the hybrids is studied using three enediols able to form charge transfer complexes: catechol, dopamine, and ascorbic acid. For each system, the process conditions driving the transition from the sol to chemical, physical, or particulate gels are explored. The structural, optical, and photoelectrochemical characterization of the amorphous hybrid materials shows analogies and differences related to the organic component. In particular, electron paramagnetic resonance (EPR) spectroscopy at room temperature reveals the presence of organic radical species with different evolution and stability, and photocurrent measurements prove the effective photosensitization of TiO_2_ in the visible range induced by interfacial ligand-to-metal charge transfer.

## 1 Introduction

The conjugation of inorganic materials with organic compounds provides emerging functional properties to the resulting hybrid materials. The study of the interaction of organic molecules with metal oxides is essential in several fields such as catalysis, photovoltaics, sensing, or drug delivery.^1,2^ Catechol (1,2-dihydroxybenzene) and its derivatives, being able to bind to almost every kind of surface (ceramics, metals, polymers, and carbon-based materials), are ubiquitous in nature, and hybrid systems containing these compounds are increasingly attractive for a bunch of applications, including adhesives (bioinspired by the sticking ability of mussels), chemo- and biosensing, imaging, and therapeutic and optoelectronic devices.^3,4^ A relevant example is the photosensitization of titanium dioxide, a wide band gap semiconductor, sought to activate visible light response. Alongside the most common dye sensitization strategy (an indirect electron transfer involving an excited state of the dye), an alternative mechanism is a direct electron transfer from the fundamental state of the sensitizer to the conduction band of the oxide, referred to as ligand-to-metal charge transfer (LMCT) or type II photosensitization. It can be induced by relatively small organic molecules that do not absorb visible light on their own.^2,5,6^ Among them, catechol is the most studied. Its interaction with TiO_2_ sols, nanoparticles, and surfaces has been investigated in detail by experiments,^7−15^ computational methods,^16^ or both.^17−21^ The electron injection upon the formation of Ti-catecholate complexes results in an absorption extended up to about 600 nm and a considerable lifetime of charge separation, which has been linked to a partial delocalization in the TiO_2_ lattice, slowing down the recombination (back electron transfer).^10^ Consequently, TiO_2_-catechol materials have shown enhanced photocatalytic activity under visible light in water splitting,^22−25^ selective oxidation of amines,^26^ Cr(VI) reduction,^24^ and inactivation of bacteria.^27^ The direct interfacial charge transfer mechanism has been investigated also for TiO_2_-based dye-sensitized solar cells.^25,28^ Moreover, catechol functionalization has been found to improve the photoresponse of other semiconductors as well, e.g., titanates.^29^

Substituted catechols, including dopamine, an important neurotransmitter, comparably affect the photoactivity of TiO_2._^14,23,30^ Experimental^9,13,31−36^ and theoretical studies^34,37−39^ focused on dopamine-functionalized TiO_2_ also reported peculiar properties, such as a favorable binding of DNA, proteins, peptides, and other biomolecules^33^ and a surface enhanced Raman scattering (SERS) effect.^38^ Moreover, dopamine and its derivatives can polymerize on TiO_2_ nanoparticles in a range of conditions:^3^ for example, oxides coupled with polydopamine show promising photocatalytic performances,^40−42^ while the *in situ* polymerization of l-DOPA (3,4-dihydroxyphenylalanine) and DHICA (dihydroxyindole carboxylic acid) produces TiO_2_-melanin hybrids with antimicrobial activity.^43−45^

The enediol functionality causes other organic compounds, even not aromatic, to show similar reactivity to catechols. An interesting example is ascorbic acid (vitamin C), which is an available, cheap, and biocompatible compound known for its efficiency as an electron donor and hence its reductant and antioxidant functions.^46^ It can act as a TiO_2_ sensitizer through LMCT^47−51^ and play a role in O_2_ reduction to generate reactive oxygen species, like superoxide radicals;^52^ nonetheless, relatively few works describe in detail TiO_2_-ascorbate hybrid systems.

In most literature reports, the preparation of TiO_2_ functionalized with organic compounds is realized by the adsorption of the molecule on the surface of crystalline (anatase or rutile) nanoparticles or films. It represents a ″top-down″ approach that can bring about physical and chemical adsorption modes. However it does not ensure an accurate control on the amount of bound organic ligand, as it depends not only on its concentration in solution but also on the accessible surface area and on the adsorption equilibrium; so in case of weak interactions, especially for large molecules with few coordinating groups, the hybrid structures may show limited stability in an aqueous environment. In addition, it is worth noting that most reports on organic-modified TiO_2_ deal with crystalline polymorphs; however, amorphous titanium oxide also revealed promising photochemical and functional properties in various applications.^53^

In this work, we propose a different ″bottom-up″ approach for the synthesis of hybrid oxides: a one-pot hydrolytic sol–gel route. Sol–gel is a versatile technique for the production of metal oxides in the form of nanoparticles, bulk gels, monoliths, or films in which additives, such as functional ligands, are uniformly mixed with the inorganic matrix at the nanoscale. Adding the ligand to the titanium precursor solution before hydrolysis and condensation reactions yields coordination complexes with modified reactivity, which, in suitable conditions, stabilize the sol or promote the growth of homogeneous chemical gels.^54,55^ Depending on the molecular structure of the ligand and on the concentration of the reagents, a variety of metal oxo-clusters can be formed, working as building blocks for metal–organic frameworks, polyoxometalates, nanostructured composites, and other hybrid materials with a specific architecture.^54,56^ Thus, the structural and morphological features of the products and the content of organic phase can be finely regulated by the process variables. We have applied such sol–gel strategy for the synthesis of TiO_2_-diketonate amorphous materials, showing unusual surface stabilization of superoxide radicals and oxidative activity in the dark.^21,57−59^ However, each complexant requires specific processing conditions to yield the desired product. A similar basic idea inspired the synthesis of crystalline organo-titania containing 4,6-dihydroxypyrimidine or *p*-phenylenediamine, with visible light photocatalytic activity.^60^ As regards catechol, a similar concept was adopted by Sugahara and co-workers, who studied the hydrolysis and condensation of Ti alkoxides modified with catechol (1:1 molar ratio).^61,62^ Anyway, their sol–gel procedure was quite complex and employed large amounts of tetrahydrofuran and aromatic solvents, and only the structural characterization of the obtained samples was reported.

We have chosen catechol, dopamine, and l-ascorbic acid for a comparative study on the sol–gel synthesis of amorphous hybrid materials based on TiO_2_ and on their structural and electronic properties, with special attention to visible light photoresponsivity. Several reaction parameters were explored: concentrations of the ligand, the titanium precursor, and water; solution pH; and the nature of the solvent. The effect of the composition and synthesis conditions on the characteristics of the interfacial charge transfer processes involved in the Ti-ligand coordinative complexes was evaluated.

## 2 Experimental Section

### 2.1 Sol–Gel Synthesis

The following reagents and solvents were used: titanium(IV) *n*-butoxide (Ti(OBu)_4_, 97+%), catechol (1,2-dihydroxybenzene, 99%), dopamine HCl (3,4-dihydroxyphenethylamine hydrochloride, 99%), l-ascorbic acid (99%), acetylacetone (*Hacac*, 2,4-pentanedione, 99+%), citric acid monohydrate (99.0%), diethanolamine (*dea*, 98%), 1-propanol (99.8+%), cyclohexane (99.5%), ethanol (99.8+%), hydrochloric acid (37 wt %), and ammonium hydroxide (28 wt %). The chemicals were provided by Sigma-Aldrich (Milan, Italy) and used as received.

In a typical hydrolytic sol–gel procedure carried out at room temperature,^59^ the precursor of the organic ligand (catechol, dopamine hydrochloride, or l-ascorbic acid) was dissolved in 1-propanol and added to the Ti precursor, Ti(OBu)_4_. The resulting solution was stirred for 30 min, and then a hydrolytic solution, containing distilled water and 1-propanol, was slowly added to the former. The composition of the reaction mixture is defined by the complexation molar ratio *c* (ligand/Ti), the hydrolysis molar ratio *h* (H_2_O/Ti), and the Ti(OBu)_4_ concentration. These parameters were varied as reported in Table 1 for selected samples and in Table S1 for all the synthesized materials. The pH of the final mixture was either left unchanged or modified up to about 4 or 10 by adding small volumes of HCl or NH_3_ to the hydrolytic solution. In some cases, a cyclohexane/1-propanol mixture or ethanol was used as the solvent. An additional ″auxiliary″ ligand (*Hacac*, *dea*, or citric acid) was tested in combination with catechol, with a molar ratio of Ti/catechol/ligand = 1:0.1:0.3. The complexation ratio 0.3 was chosen as it is the lowest at which all three ″auxiliary″ ligands alone induced relatively fast gelation in the adopted conditions. This ligand was added first to Ti(OBu)_4_ followed by catechol.

**Table 1:** Synthesis Conditions of the Most Deeply Characterized Hybrid TiO_2_ Samples, Obtained as Chemical Gels, with Catecholate (cat), Dopamine Anion (dop), or Ascorbate (asc) as Ligands

sample	*c* = ligand/Ti^a^	[Ti] (mol/L)	*h* = H_2_O/Ti^a^	solvent	additives	gelation time
T-cat0.05	0.05	0.57	2	1-propanol/cyclohexane	HCl	1 h
T-cat0.1	0.10	0.57	2	1-propanol/cyclohexane	HCl	1 day
T-dop0.05	0.05	0.52	4	1-propanol		15 min
T-dop0.1	0.10	0.38	4	1-propanol		2 days
T-asc0.05	0.05	0.30	4	1-propanol	HCl	7 days
T-asc0.1	0.10	0.30	4	1-propanol	HCl	3 days

a Molar ratio.

After the addition of the hydrolytic solution, the systems showed precipitation or gelation in variable times, depending on the conditions. More details are reported in Table S1. The chemical or physical wet gels (Figure S1) were left aging for at least 1 day and dried in air at 60 °C until constant weight. Finally, the hybrid xerogels were ground before characterization. The samples are named indicating the ligand and its nominal content; samples obtained by precipitation instead of homogeneous gelation are denoted by the final letter ″p″, and the mixed samples containing *Hacac*, citric acid, and *dea* are denoted by the final letter ″A″, ″C″, and ″D″, respectively.

### 2.2 Physicochemical Characterization

Fourier Transform infrared (FTIR) spectra were recorded using a Nicolet 5700 FTIR spectrometer (Thermo Fisher, Waltham, MA, USA) equipped with a DTGS KBr (deuterated triglycine sulfate with potassium bromide windows) detector. The transmittance spectra were acquired mixing the sample in KBr pellets, recording 32 scans with a resolution of 2 cm^–1^.

Thermogravimetric and differential thermal analysis (TG-DTA) was performed by an SDT Q600 simultaneous thermoanalyzer (TA Instruments, New Castle, DE, USA), heating in air at a 10 °C min^–1^ rate.

Ultraviolet–visible–near-infrared diffuse reflectance (UV–vis–NIR DRS) spectra were recorded on a Shimadzu UV-2600i double beam spectrophotometer with an ISR-2600Plus two-detector integrating sphere (Shimadzu, Japan) using BaSO_4_ as standard.

Electron paramagnetic resonance (EPR) spectra of the samples were recorded using an X-band (9 GHz) Bruker Elexys E-500 spectrometer (Bruker, Rheinstetten, Germany). The measurements were performed at room temperature, collecting 16 scans, with the following instrumental settings: sweep width, 140 G; resolution, 1024 points; modulation frequency, 100 kHz; modulation amplitude, 1.0 G; time constant, 20.5 ms; and attenuation, 10 dB. The *g* factor value and the spin density of the samples were evaluated by means of an internal standard, Mn^2+^-doped MgO, and calibrated with reference to a diphenylpicrylhydrazyl (DPPH) standard solution. Line fitting of the EPR spectra was performed on the Bruker Xepr software.

Cyclic voltammetry (CV) and photocurrent measurements were performed using a photoelectric spectrometer (Instytut Fotonowy, Krakow, Poland) and a three-electrode configuration, with Ag/AgCl as the reference electrode and platinum wire as the counter electrode. A thin layer of the material (the working electrode) was deposited at the surface of an ITO-coated transparent PET foil (60 Ω/sq resistance, Sigma-Aldrich). The sample (15 mg) was finely ground in the agate mortar with a few drops of water. The formed suspension was casted (the so-called doctor blade method) on the surface of the ITO-coated transparent PET foil. The deposited uniform film was then dried under flowing air at *ca*. 60 °C. The electrolyte (0.1 mol L^–1^ KNO_3_, pH = 6.1) was purged with argon for 15 min prior to and during the measurement. CV scans were acquired in the dark, with a 10 mV/s scan rate. Photocurrents were recorded irradiating the working electrode from the backside with a xenon lamp in the range of 330–550 nm with 10 nm step, applying voltages in the range between −0.2 and 1.0 V (vs Ag/AgCl). The size of the working electrode is determined by the diameter of the window (1.0 cm), so the area of the irradiated surface is *A* = 1/4π cm^2^ = 0.785 cm^2^.

## 3 Results and Discussion

### 3.1 Synthesis of the TiO_2_-Enediol Hybrid Materials

In the sol–gel synthesis of a metal oxide, the addition of a complexing compound to the metal precursor allows tuning the rates of hydrolysis and polycondensation, driving the system toward the formation of stable sols, bulk gels, or small particles.^54,55^ The rate, extent, and mechanism of these reactions depend on different factors, including the structure of the complexing ligand, its concentration relative to the metal, the amount of water, the properties of the solvent, and the solution pH, which in turn determine the structural properties of the products.^56,63,64^ We explored the evolution of hybrid sols, i.e., the colloids produced by hydrolysis and partial condensation of titanium alkoxide species modified with the enediol ligands, with the aim to understand the conditions promoting homogeneous gelation or precipitation.

#### 3.1.1 TiO_2_-Catechol

**Chart 1:**
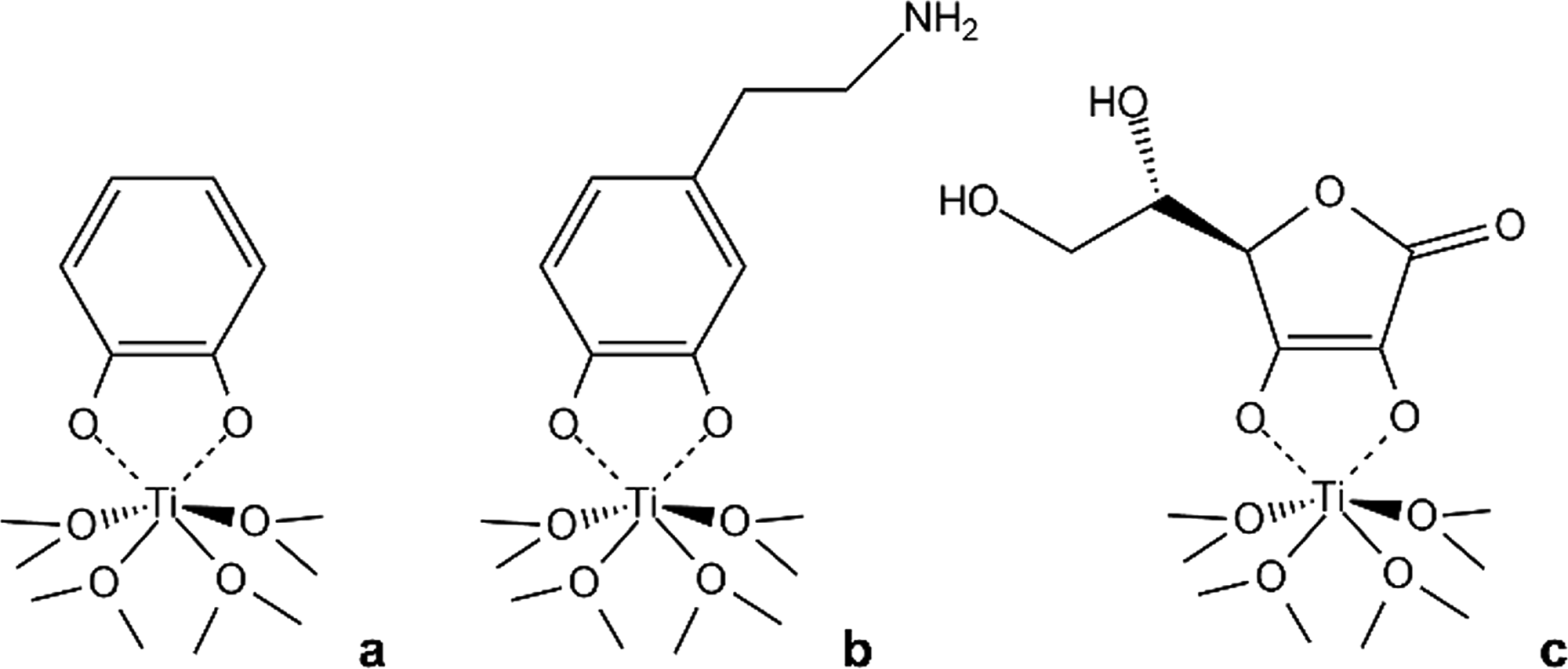
Possible structure of the Ti(IV)-ligand complexes in the TiO_2_-based hybrid materials containing catechol (a), dopamine (b), and l-ascorbic acid (c)

In the Ti-catechol system, several reaction parameters were studied: the concentration of catechol, Ti(OBu)_4_, and water (then the complexation ratio (ligand/Ti) *c* and the hydrolysis ratio (H_2_O/Ti) *h*); pH; and solvent (see Table S1). When the catechol solution was added to Ti(OBu)_4_, an orange to dark red coloration immediately appeared, depending on their relative concentration, attesting to the formation of the Ti-catecholate (Ti-*cat*) complex. The coordination of catechol and dopamine on small TiO_2_ nanoparticles was found to be associated with large binding constants,^9,13^ and upon modification of Ti alkoxides with *cat*, a considerable stability of the complex to hydrolysis was reported.^62^ Aiming to obtain uniform gels that retain the ligand bonded to the oxide matrix, we tried to moderate the hydrolysis and condensations rates using low *h* values. Anyway, in all the tests run in 1-propanol solvent, after the addition of the aqueous solution, the reaction mixture became turbid and a precipitate or a gel-like viscous mass formed, in shorter or longer times. We recently observed such behavior for *c* = 0.2.^21^ Varying *c* in the range from 0.01 to 0.4 slightly affected the outcome, as well as a reduction of *h* from 4 to 2, a dilution of the mixture ([Ti] from 1.0 to 0.3 mol L^–1^), or a pH variation by adding HCl or NH_3_, which only delayed precipitation.^59^ The behavior of this system may be explained by the structure of *cat* coordinated to Ti ions in the heteroleptic complexes, exposing the benzene ring outward (see Chart 1). When the catechol content is relatively high, the hydrophobic coverage on the primary particles of the sol increases, and in a polar solvent like propanol, they become susceptible to gradually aggregate and precipitate, even though in certain conditions the separation from the solvent is not marked and a gel-like mass is formed, which can be described as a physical gel with loose bonds between the clusters. With a lower complexation ratio, the hydrophobicity is reduced, but the ligand is unable to properly modulate the condensation rate and prevent the formation of particles large enough to show phase separation.

The challenge of achieving TiO_2_-*cat* chemical gels was faced by two different strategies: the use of a mixed solvent with a lower polarity than pure propanol and the addition of a second ″auxiliary″ ligand, which induces gelation in similar conditions. The introduction of cyclohexane (with a cyclohexane/propanol 2:1 volume ratio) to reduce the polarity of the reaction environment yielded uniform gels, closer to a chemical gel than those obtained in pure propanol. With *c* = 0.05, a rather opaque gel formed in less than 1 h, while with *c* = 0.1, a more limpid gel formed in about 24 h (Figure S1). The gelation process strongly depends on the interactions between the primary particles composing the sol and, in the presence of surface ligands, on the interaction between these ligands and the solvent.^55^ As predicted, a less polar solvent increased the solubility of the Ti alkoxide/hydroxide clusters capped by *cat*, allowing their controlled aggregation and the growth of a cross-linked network (gel), while an increase in the ligand concentration reduced the rate of this process.

Concerning the second mentioned approach, the idea was also to investigate a mixed hybrid system, including two different organic ligands. Three complexing compounds with different functionality and acid/base character were chosen for a comparative purpose: acetylacetone (*Hacac*), a β-diketone commonly used as a stabilizer in sol–gel processing; citric acid, a tricarboxylic acid and effective chelating agent for several metal ions; and diethanolamine (*dea*), a potentially tridentate ligand, particularly used in the stabilization of TiO_2_ sols for coatings.^54,65^ The modification of Ti alkoxides with such molecules, in particular *Hacac* and carboxylic acids, acting as bidentate chelators, has been widely studied from the viewpoint of the oxo-clusters formed in the solution^56^ and of the derived hybrid materials, which often exhibit a porous network structure.^57,63,64^ Introducing catechol after the additional ligand always caused the light-colored solution to turn intense red, showing that Ti-*cat* complexation was not hindered. In all three cases, gelation was accomplished (see Table S1). The Ti-*cat*-*acac* system appeared to be the most sensitive to the reaction parameters and required their optimization and the tuning of pH. A homogeneous gel (T-cat0.1A) was obtained by adding first a small amount of HCl (0.1 mol L^–1^) in the aqueous solution (*h* = 4, pH ∼4), to assist hydrolysis forming a stable sol, and subsequently NH_3_, increasing pH to about 10, to catalyze polycondensation. The other two mixed systems with citric acid (T-cat0.1C) and *dea* (T-cat0.1D) required a higher dilution ([Ti] = 0.5 mol L^–1^) and less water (*h* = 2) to yield uniform opaque gels, dark red and dark orange, respectively, in about 10 min. These mixed hybrid gels are expected to own different structural features due to the different pH and nature of the additional ligands. Acidic conditions are known to favor the growth of linear chains during polycondensation and eventually gelation, while basic conditions tend to promote more branched and dense structures, hence the precipitation of small particles. Interestingly, here gelation was achieved also at basic pH (T-cat0.1A and T-cat0.1D), which can be explained by the relatively high concentration of ligands, blocking coordination sites on Ti^4+^ ions, and the favorable interaction of these polar ligands with the alcohol solvent. Higher complexation ratios and a larger number of coordinating groups, as in citrate, are expected to decrease the degree of condensation, driving the formation of open porous structures.

#### 3.1.2 TiO_2_-Dopamine

The aminoethyl group of dopamine provides it a remarkably different reactivity compared to catechol, which was reflected in the behavior of the Ti-dopamine system. A dark red, limpid, and homogeneous chemical gel formed in 1-propanol solvent at neutral pH (see Table 1). The gelation occurred in 15 min with *c* = 0.05, while with *c* = 0.1, it was much slower (about 2 days). Dopamine in bidentate coordination through the diol moiety has the amino group (mainly protonated at pH about 7) available to interact with another Ti atom, with the solvent, or with another dopamine molecule through hydrogen bonds or an acid/base reaction. Since dopamine polymerization can be initiated in a basic environment,^3,40^ we checked the effect of NH_3_ addition in the hydrolytic solution (pH ∼10). The result was the fast precipitation of a dark orange powder, in accordance with the usual influence of the base on the nucleophilic substitution reactions, inducing faster and branched condensation. The observation that a relatively small amount of dopamine can readily promote the gelation of TiO_2_ could be extended to similar catechol-like compounds and facilitate the sol–gel preparation of functionalized hybrid coatings without the need for other stabilizers.

#### 3.1.3 TiO_2_-Ascorbic Acid

The addition of an ascorbic acid solution to Ti(OBu)_4_ induces a dark red coloration as well, proving the complexation. With *c* = 0.1 or 0.2 and [Ti] ≥ 0.5 mol L^–1^, immediate and incomplete gelation occurred, leading to non-uniform gel-like products. When the mixture was diluted to [Ti] = 0.3 mol L^–1^ and HCl (0.1 mol L^–1^) was introduced with the hydrolytic solution, a slower and ″cleaner″ gelation occurred (see Table 1). It is interesting to note the inverse dependence of the gelation time on the complexation ratio in this case: T-asc0.05 (about 7 days) > T-asc0.1 (about 3 days) > T-asc0.2 (few hours). It is an opposite trend compared to the usual one, i.e., an increase of the gelation time with the ligand concentration, as observed here with catechol and dopamine and previously reported for acetylacetone.^63^ As the complexation of ascorbate is supposed to act primarily through the enediol moiety, a possible explanation could be in the additional interactions, e.g., the coordination of another Ti atom by the free hydroxyls of ascorbate, or a hydrogen bonding with another alkoxide oligomer, facilitating condensation reactions and gelation. Moreover, it was demonstrated that the binding of Ti^4+^ by ascorbate is strong enough to prevent hydrolytic precipitation but weaker than binding by other common biological ligands such as citrate;^66^ therefore, the possible mobility of the ascorbate ligands can also be considered in the equilibria established varying their concentration.

The effect of solvent was also examined. Despite the slightly higher solubility of ascorbic acid in ethanol than in 1-propanol, during water addition, fast precipitation occurred in the ethanol solvent, while identical conditions in 1-propanol allowed a slow sol–gel transition, which is likely related to the faster exchange of ethoxide groups substituted on the Ti complexes and oligomers compared to propoxide ones. In summary, the TiO_2_-ascorbate system has a multifaceted behavior and can be directed to different kinds of product (chemical, physical, or particulate gels).

### 3.2 Structural Properties

**Figure 1:**
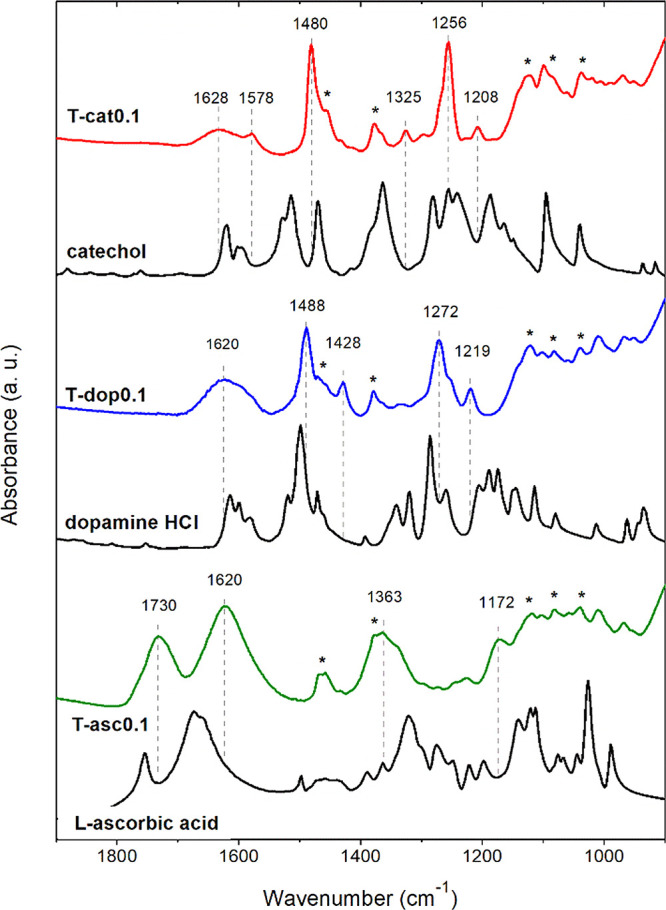
FTIR spectra of representative hybrid xerogels and of the organic compounds used as ligands. The asterisks (*) indicate bands due to residual alkoxide groups.

All the studied materials, both particulate and chemical gels, are amorphous, as attested by XRD profiles (Figure S2). Infrared spectroscopy offers information on the type of binding between the organic ligands and the TiO_2_ matrix, which is crucial in determining the electronic coupling and charge transfer characteristics of the complex.^16,20^ The most relevant range of the FTIR spectra of representative samples and pure complexing molecules is shown in Figure 1 (see Figure S3 for the full range). All the hybrid xerogels exhibit Ti–O vibration bands below 800 cm^–1^; an intense broad band of O–H stretching around 3000–3500 cm^–1^, indicating considerable surface hydroxylation; and a band about 1620 cm^–1^ due to bending in adsorbed water molecules.

The vibrational spectrum of free catechol includes several bands, mainly due to the stretching of the aromatic ring and of the phenol groups (C–O) and the bending of O–H and C–H bonds. Upon complexation, a modification of the spectrum is evident: the two strongest bands at 1480 and 1256 cm^–1^ are ascribed, respectively, to the stretching of the C–C bonds in the aromatic ring and of C–O groups involved in the coordination.^9,11,12,14,30^ The charge delocalization related to the formation of the complex affects also the stretching vibrations in the aromatic ring, causing a shift of some less intense bands. Those at 1578 and 1625 cm^–1^ are associated with combinations of stretching modes, and the one at 1208 cm^–1^ is associated with C–H and O–H bending modes.^12,30^ Samples obtained by precipitation and gelation present analogous spectra, with absorbance intensities increasing with the nominal catechol/Ti ratio (Figure S4). Catechol coordination to Ti^4+^ ions in the solution and its adsorption on TiO_2_ nanoparticles have been the object of spectroscopic and computational studies. It is established that its bidentate coordination preferentially occurs by a dissociative mechanism through both deprotonated hydroxyl groups, i.e., as catecholate (*cat*) anion; however, it is not easy to discriminate between a chelating and bridging geometry (forming a five- and seven-atom ring, respectively), as the predominant geometry may depend on different factors, such as the type of adsorption site (crystal facet, edge, terrace, or point defect).^8,15^ Mixed geometries are also possible, as the most stable one calculated on the O-defective anatase (101) surface, namely a bidentate mode with one oxygen of *cat* coordinating two adjacent Ti atoms.^21^ The wavenumbers of the main bands observed in Figure 1 are close to those measured for the Ti(*cat*)_3_^2–^ complex in the solution^11^ and intermediate between those predicted for bridging and chelating the TiO_2_-*cat* surface complexes.^30^ In our materials, the complex forms on the monomeric Ti(IV) alkoxide precursor in the first step of the synthesis procedure, so the binding of *cat* should be chelating. Then, during the structuring of the oxide matrix, a distribution of binding modes might be obtained, considering the coordination equilibria and the mobility across the TiO_2_ surface observed for *cat*.^17^

The addition of a second ligand (*Hacac*, citric acid, or *dea*) during the TiO_2_-catechol synthesis, promoting gelation in the alcohol solvent, makes the features of both molecules discernible in the FT-IR spectra (Figure S4). The two major bands of bonded *cat* are unchanged, while the others are covered by the bands of the second ligand. The T-cat0.1A spectrum shows bands typically ascribed to the Ti-*acac* chelate ring in TiO_2_-*acac* materials,^21,57^ the only significant variation being the appearance of a band centered at 1400 cm^–1^, absent in the hybrids with *acac* or *cat* alone, suggesting some interaction between the two molecules. The T-cat0.1C spectrum carries evidence of coordinated carboxylate groups of citrate, possibly with a bidentate bridging geometry, while that of T-cat0.1D shows characteristic features of *dea*.^65^

Dopamine is expected to behave similarly to catechol in the coordination on TiO_2_. However, analogies and differences are revealed between the spectra of T-dop0.1 and T-cat0.1 (Figure 1). The main bands have similar relative intensities, and the two strongest bands are found at slightly higher frequencies, 1488 and 1272 cm^–1^, again due to the stretching of aromatic C–C bonds and of C–O bonds.^32,35^ These wavenumbers are about 10 cm^–1^ lower compared to both free dopamine and dopamine adsorbed on TiO_2_,^35,36^ attesting a strong coordination bond. In analogy with catechol, the bidentate coordination of deprotonated dopamine is favored but a clear prevalence of the bridging or chelating geometry has not been demonstrated.^37,38^ The overlapped bands between 1620 and 1585 cm^–1^ may collect contributions from aromatic ring stretching and asymmetric bending of N–H, besides adsorbed water. The new band at 1428 cm^–1^ can be assigned to a symmetric N–H umbrella mode characteristic of the protonated NH_3_^+^ group of dopamine.^35^ In fact, recent calculations revealed that the amino group has a relevant role in dopamine interaction with TiO_2_, being able to coordinate a surface Ti atom, and that its protonation is favored at high surface coverage.^39^

The IR spectrum of ascorbic acid displays characteristic bands, among them the stretching of the lactone C=O at 1755 cm^–1^ and of C=C around 1665 cm^–1^, a concerted ″semicircle stretch″ mode at 1320 cm^–1^, and the O–H vibrations of hydroxyls between 3220 and 3520 cm^–1^.^47^ The T-asc0.1 xerogel exhibits marked shifts of most bands, indicative of a bidentate complexation, which should occur through the enediol group, favored by resonance of the deprotonated structure. The charge delocalization involves shifts to lower frequencies of ν(C=O) and ν(C=C), showing up at 1722 and 1616 cm^–1^, while the vibration of the ring appears shifted to 1363 cm^–1^.^47^ The stretching of coordinated C–O groups is associated to the bands at 1172 cm^–1^ and lower wavenumbers.^67^ The results are consistent with chelating binding, forming a five-membered ring around the surface Ti atoms, with a favorable conformation of bond angles and distances for octahedrally coordinated Ti. The observed shifts seem larger than those recorded by Rajh et al.,^47^ suggesting that also the Ti-ascorbate complexation obtained by our sol–gel procedure may be stronger than that realized by surface adsorption.

**Figure 2:**
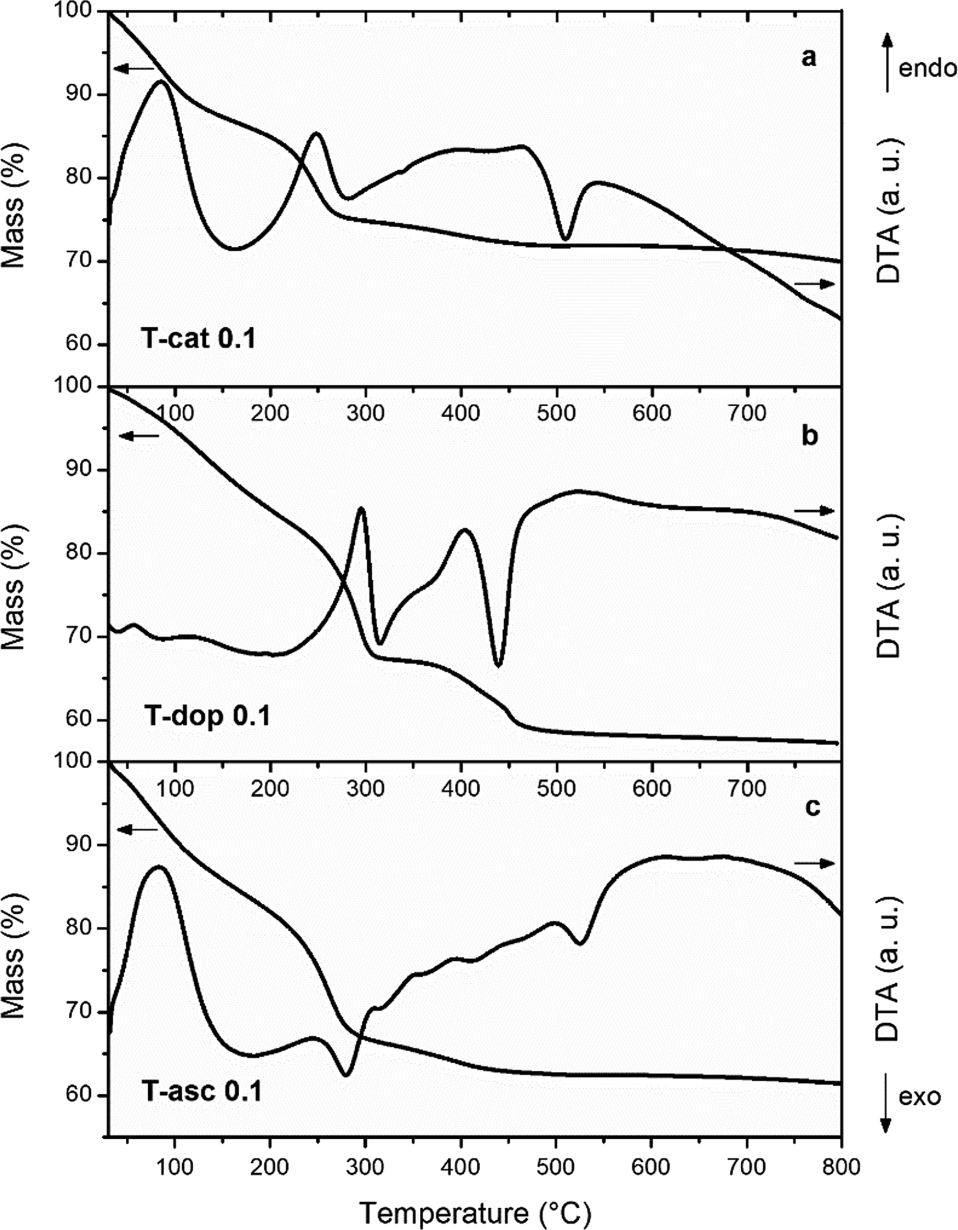
TGA and DTA profiles of hybrid xerogels T-cat0.1 (a), T-dop0.1 (b), and T-asc0.1 (c) recorded in N_2_ at a 10 °C/min heating rate.

Thermal analysis provides information on the stability of the hybrid oxides with temperature and allows an estimate of the amount of adsorbed and organic species in the structure. The TG-DTA profiles of representative samples are shown in Figure 2. The overall mass loss for T-cat0.1, T-dop0.1, and T-asc0.1 is about 30, 42, and 38 wt %, respectively. The loss below 200 °C (around 15 wt %) is due to the vaporization of adsorbed water and residual solvent molecules. The main mass decrease, again associated with an endothermic DTA peak, is centered at 250 °C for T-cat0.1 and T-asc0.1 and 290 °C for T-dop0.1, and is attributed to the volatilization of most of the organic ligand. However, residual alkoxide groups, not completely hydrolyzed before polycondensation, likely contribute to the mass decrease in this temperature range, as well as the dehydroxylation of the surface.^30,61^ The mass loss is concluded in the range 350–450 °C, with the removal of the products of partial pyrolysis or polymerization of the complexing ligands in the structure. The exothermic effect seen at 440 °C for T-dop0.1 and above 500 °C for the other two samples is ascribable to the crystallization of the amorphous TiO_2_ matrix. Samples obtained with different amounts of catechol were also analyzed by TG-DTA (Figure S5). Although the theoretical catechol content in T-cat0.01, T-cat0.1, and T-cat0.4 is 1.5, 12, and 35 wt %, respectively, the overall mass loss is comparable for all of them (30–35 wt %), confirming that the evacuation of residual alkoxide ligands and alcohol molecules can cause a relevant fraction of the mass losses recorded in the 250–280 °C range. However, the mass losses above 300 °C are proportional to the catechol content and spread up to higher temperature, consistent with a larger amount of carbonaceous residues. In the sample with the lowest catechol fraction, crystallization is free to occur at lower temperatures, as attested by the narrow exothermic DTA peak at 390 °C.

### 3.3 Optical Properties

**Figure 3:**
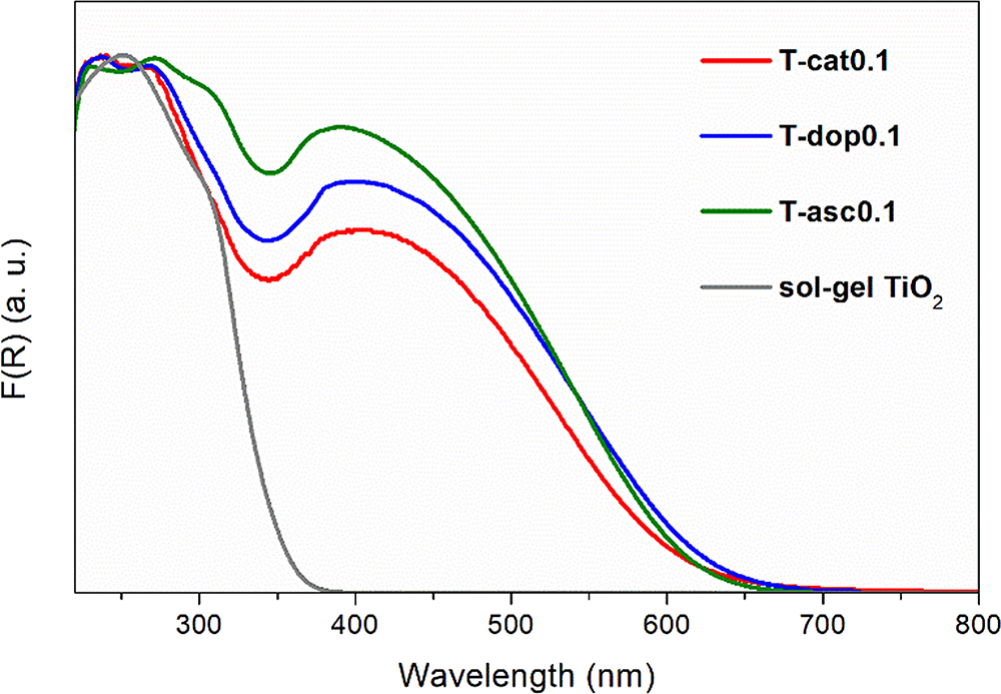
UV–vis DR spectra of representative hybrid xerogels, reported as a normalized Kubelka–Munk function of reflectance, along with an amorphous TiO_2_ sample prepared by sol–gel as reference.

Diffuse reflectance UV–visible spectra (Figure 3) evidence that the dark red coloration of the hybrid samples (Figure S1) is reflected in a large red shift of the absorption edge compared to bare TiO_2_, with tails reaching 700 nm. The three organic molecules are colorless: free catechol, dopamine, and ascorbic acid present a π–π* transition (HOMO–LUMO) between 265 and 280 nm and no absorption above 300 nm.^9,48^ The bands appearing in the visible range arise from an interfacial charge transfer, a direct excitation of an electron from the HOMO of the ligand (a π orbital) to the Ti 3d orbitals, namely, a ligand-to-metal charge transfer (LMCT), better defined as a ligand-to-band CT when the injected electron is delocalized in the conduction band instead of being trapped on a Ti^4+^ ion. The partially delocalized nature of the catechol-TiO_2_ CT was predicted theoretically^16,21^ and corroborated by ultrafast spectroscopic measurements,^10,68^ although some results described it as rather localized.^69^ Similar resulting absorption spectra were reported for surface-modified nanoparticles or films.^9,69^ Here the relative intensities of the CT bands are comparable to those of the band gap transition, suggesting the presence of a large concentration of active complexes throughout the surface and likely the bulk of the materials. For a TiO_2_-*cat* sample with a higher ligand content (*c* = 0.2), a broader absorption stretching into the NIR range was observed,^21^ suggesting a broader and more disordered distribution of electron states. The CT bands are centered around 420 nm, close to the values reported in the literature for catechol- and dopamine-modified TiO_2_ (about 400–420 nm).^7,8,13^ In these hybrid systems, the band gap energies evaluated from UV–vis DR data (Figure S7) refer to the energy difference between the conduction band edge and the HOMO of the ligand, so they can be considered as ″apparent″ or ″effective″ band gaps resulting from the CT complex.^9^ TiO_2_ is an indirect band gap semiconductor; however, it is not straightforward to determine if the effective gaps of TiO_2_-based hybrids are better described as direct or indirect. Tauc plot elaboration was performed for both cases (Figure S7). The values estimated for indirect band gap are between 1.8 and 1.9 eV, and those for direct band gap are between 2.2 and 2.3 eV. Taking into account the determined energies and the spectra presented in Figure 3, the indirect band gap better describes the synthesized materials, characterized with the band gap energies of 1.8–1.9 eV corresponding to the wavelengths of 650–690 nm.

The strong electronic coupling between *cat* and titanium allows an efficient electron injection under relatively low energy radiation.^49,68^ An analogous situation can be depicted for dopamine and ascorbate, considering the analogies in their molecular structure, in the coordinative interaction with Ti ions and in the energy levels (see also the CV data, Section 3.5). The holes generated by such charge transfer are preferentially localized on the organic ligand, as can be inferred from the EPR results.

### 3.4 Paramagnetic Properties

**Figure 4:**
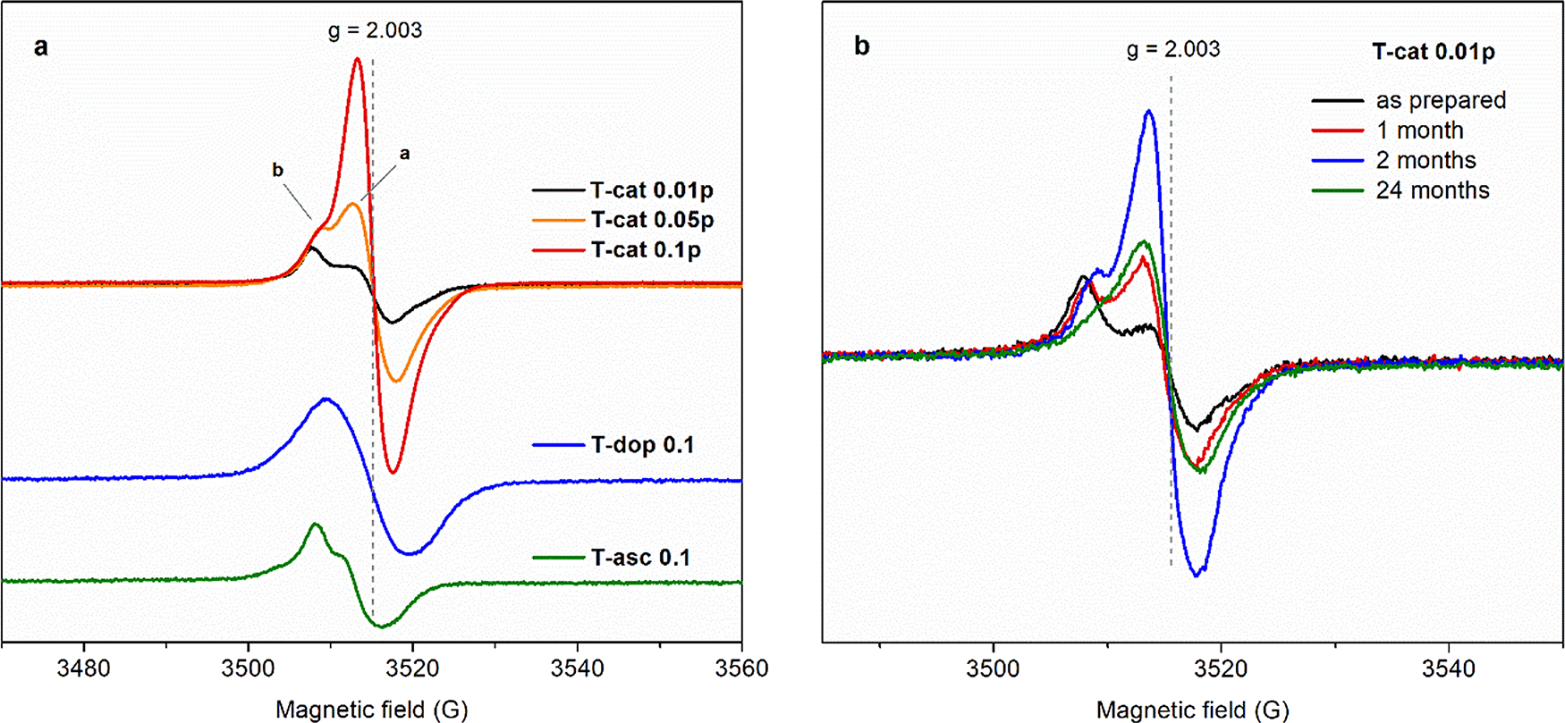
EPR spectra recorded at room temperature on representative hybrid samples (a) and on the T-cat0.01p sample as prepared and after storage for different times in ambient conditions (b).

EPR spectra representative of the studied solid systems are displayed in Figure 4a. Interestingly, TiO_2_-*cat* materials present a composite signal: a single peak (indicated as ″a″) centered at *g* factor ∼2.003 and an overlapped peak (indicated as ″b″) at a lower field, with *g* ∼2.006, as confirmed by line fitting (see Figures S8–S10). As the catechol/Ti molar ratio increases, the overall intensity of the signal increases, showing that it is actually associated with the ligand, and the relative intensity of the two components changes as described below, suggesting that the two peaks are related to different paramagnetic species. The asymmetric signal of TiO_2_-*asc* seems to comprise a double component too, while TiO_2_-*dop* exhibits a rather broad and symmetric peak.

Few works described the EPR analysis of such hybrid materials. Studies on the radical species formed by catecholic compounds and their derivatives interacting with the surface of TiO_2_ and other metal oxides reported a variability of EPR signals. Rajh and co-workers investigated TiO_2_ nanoparticles modified by surface adsorption of various molecules, including ascorbic acid,^47^ catechol,^9^ and dopamine.^9,33^ Recording EPR spectra on aqueous colloids at 4 K, under visible light illumination, they identified the charge carriers resulting from the excitation of the CT complex: the electron trapped at Ti^3+^ centers, which are however hardly detected at room temperature, and the hole localized on the organic ligand, associated with a broad singlet, with *g* values of 2.0036, 2.0038, and 2.0040–2.0049 for dopamine, catechol, and ascorbate, respectively, and a peak-to-peak line width (Δ*B*) of about 10 G. This kind of signal strongly resembles those recorded on our hybrid materials (Figure 4a), with an excellent agreement in *g* values and some differences in the line width: here Δ*B* values range from 6–7 G for T-*cat* and T-*asc* samples (considering peak ″a″) to 10 G for T-*dop* gel (Table 2). The peak width was reported to broaden with the size of the ligand due to the coupling of H and other atoms with the unpaired electron, while the variability in *g* values was attributed to the number of π electrons in the molecule.^9^ A similar spectrum, with *g* = 2.0033 and Δ*B* = 6 G, was reported for a TiO_2_ film surface-modified with catechol and was attributed to a stable semiquinone radical anion produced by catechol oxidation through a CV scan.^22^ Dellinger and co-workers, analyzing the persistent free radicals produced by catechol pyrolysis on CuO and Fe_2_O_3_, found a ″split″ signal similar to ours.^70^ They assigned the peak with *g* 2.004–2.006 to *o*-semiquinone, while its dimer, rather than an isomer, another decomposition product, or a single electron trapped in oxygen vacancies, was proposed as the source of the component with a lower *g* value.^70,71^ Indeed, the singlet assigned to such oxygen vacancies is supposed to have a *g* of about 2.003, while a *g* of about 2.004 was reported for the products of catechol autoxidation.^72^ However, considering the trends in the relative intensities of the two observed components, the attribution of both to different catechol-derived radicals, produced by successive oxidation steps, seems more reliable.

**Table 2:** Parameters of the EPR Signals of the Hybrid Samples as Synthesized^a^

sample	g factor (±0.0003)	EPR peak width (G) (±0.2)	spin density (g^–1^) (±10%)
T-cat0.05	2.0031 (a), 2.0062 (b)	6.5 (a), 3.5 (b)	2.5 × 10^16^
T-dop0.05	2.0034	9.9	1.5 × 10^16^
T-asc0.05	2.0045 (a), 2.0065 (b)	7.5 (a), 2.3 (b)	1.0 × 10^16^

a Data related to peaks ″a″ and ″b″ are reported.

The existence of a narrower overlapped signal appears evident also in TiO_2_-*asc* (although a small axial *g*-anisotropy was predicted for this system^47^), while in TiO_2_-*dop*, it could be hidden behind the broader Gaussian singlet, which gives a satisfactory fitting of the spectrum (see Figure S8).

We noticed an evolution of the signals in time, which was particularly interesting for TiO_2_-*cat*. EPR spectra recorded on T-cat0.01p after storing the sample under ambient conditions for 1, 2, and 24 months (Figure 4b) revealed a clear increase of the overall intensity within the first months followed by a slow decline. Meanwhile, the two components showed an opposite trend: the intensity of peak ″a″ initially increased, while that of peak ″b″ gradually decreased. These trends are displayed in Figure S9 and the corresponding fitted spectra in Figure S10. They could be explained by the occurrence of a first oxidative oligomerization process of some free or released catechol molecules, leading to the formation of small oligomers,^11,24^ which are associated to a higher content of carbon-centered radicals (peak ″a″).^43,73,74^ Then, the autoxidation process induced by TiO_2_^75^ together with the prolonged exposure to environmental conditions could induce a slow stepwise degradation of the organic ligand, causing a loss of the EPR signal intensity.

To verify this hypothesis, FTIR spectra were recorded on samples after prolonged storage (Figure S11). T-cat0.1 after 1 year showed an almost unmodified spectrum, while T-cat0.01p after 2 years revealed evident changes in most of the main IR bands, including the reduction of the relative intensities, slight shifts, and the growth of some bands (e.g., at 1380 cm^–1^) resembling those observed in free catechol or in photopolymerized catechol ligands on TiO_2._^11^ It could be inferred that, in the studied conditions, a limited fraction of the organic ligands contributes to the redox equilibria that generate the detected radical species; thus, the chemical transformations are more evident in the sample with the lowest organic content (T-cat0.01p).

Contrary to TiO_2_-*cat*, in samples with dopamine and ascorbate, the total signal intensities decreased already 1 month after the synthesis by about 50% in T-dop0.05 and 90% in T-asc0.05 (Figure S8). EPR spectra recorded after 1 year on these systems confirmed the trends (data not shown). The corresponding FTIR spectra (Figure S11) showed negligible changes in TiO_2_-*dop* and more noticeable alterations in a TiO_2_-*asc* sample, in accordance with the faster decay of the EPR signal intensity for the latter.

These observations suggest that the studied interfacial CT complexes are dynamic in time and involve the formation of different radical species with different stability, possibly depending also on the interaction of the sample with ambient light and adsorbed species.

Multicomponent signals appear also in mixed-ligand hybrids (Figure S12). In T-cat0.1D, an increase in intensity and width is particularly evident. Here an interplay of catechol with other adjacent ligands, hence a wider distribution of the radical centers, may justify the broadening of the signals. Furthermore, it can be noticed that the anisotropic signal of the superoxide radical anion (O_2_^•–^) is not spotted in any of those spectra, although we previously proved the ability of TiO_2_-*acac* hybrid xerogels to spontaneously generate and stably adsorb superoxide on their surface in contact with air^57,58^ and we observed this phenomenon on TiO_2_-citrate.^59^ Conversely, DFT calculations concluded that, on a TiO_2_-*cat* surface, the same process is not energetically favored.^21^ The presence of *cat* apparently inhibits the formation or stabilization of superoxide radicals, promoted by *acac* and citrate.

In summary, it seems reasonable to attribute the EPR signal of the hybrid samples to organic radicals produced by electron transfer to the oxide. It is worth emphasizing that the EPR spectra were collected at room temperature without continuous light irradiation or any other kind of activation of the as-prepared materials besides the exposure to ambient light. It can be deduced that the strong chemical bonding and the related electronic coupling obtained in these systems generate an extended and stable charge separation, with holes localized on the enediol ligands, forming persistent radicals.

### 3.5 Photoelectrochemical Properties

**Figure 5:**
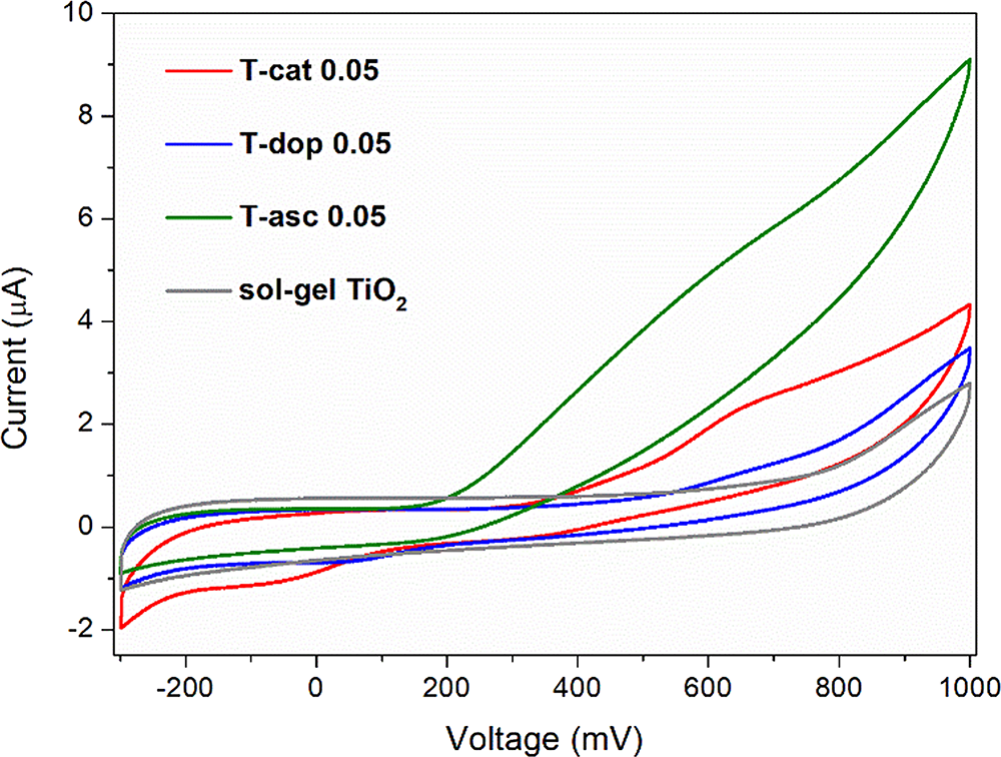
Cyclic voltammograms recorded on the PET/ITO electrode covered with hybrid xerogel powders and a reference TiO_2_, in 0.1 M KNO_3_ aqueous electrolyte saturated with Ar, at a scan rate 10 mV/s. For the sake of clarity, only single cycles are presented. Reference electrode: Ag/AgCl.

The occurrence of oxidation and reduction phenomena in a semiconducting material can be analyzed by cyclic voltammetry (CV), which provides indications on the potential of surface electron states.^31^ The electrochemical characterization was performed on xerogel powders with a 0.05 ligand/Ti molar ratio to assess the effect of a relatively low organic content. A CV profile recorded in the dark for each sample is shown in Figure 5. Alongside the typical behavior of TiO_2_, the hybrid samples exhibit a reproducible oxidation peak: this is clearly visible in T-cat0.05 and T-asc0.05, centered at about 650 and 600 mV (vs Ag/AgCl), respectively, and can be individuated in T-dop0.05 as a slope change around the same potential. The data are in agreement with those reported in the literature for TiO_2_ modified with catechol^22,27^ and ascorbic acid,^48^ ascribed to their oxidation to *o*-semiquinone and ascorbate radicals. These species, as well as dopamine semiquinone, can undergo also a second oxidation step to *o*-benzoquinone and dehydroascorbic acid, respectively. T-cat0.05 and T-dop0.05 also exhibit a reduction peak, at about −100 and 50 mV, respectively, indicating that ligand oxidation equilibrium can be at least partially inverted, although the large peak separation (Δ*E*) suggests a high resistance to the reduction. The width and low intensity of the observed redox processes could be due to slow kinetics.

**Figure 6:**
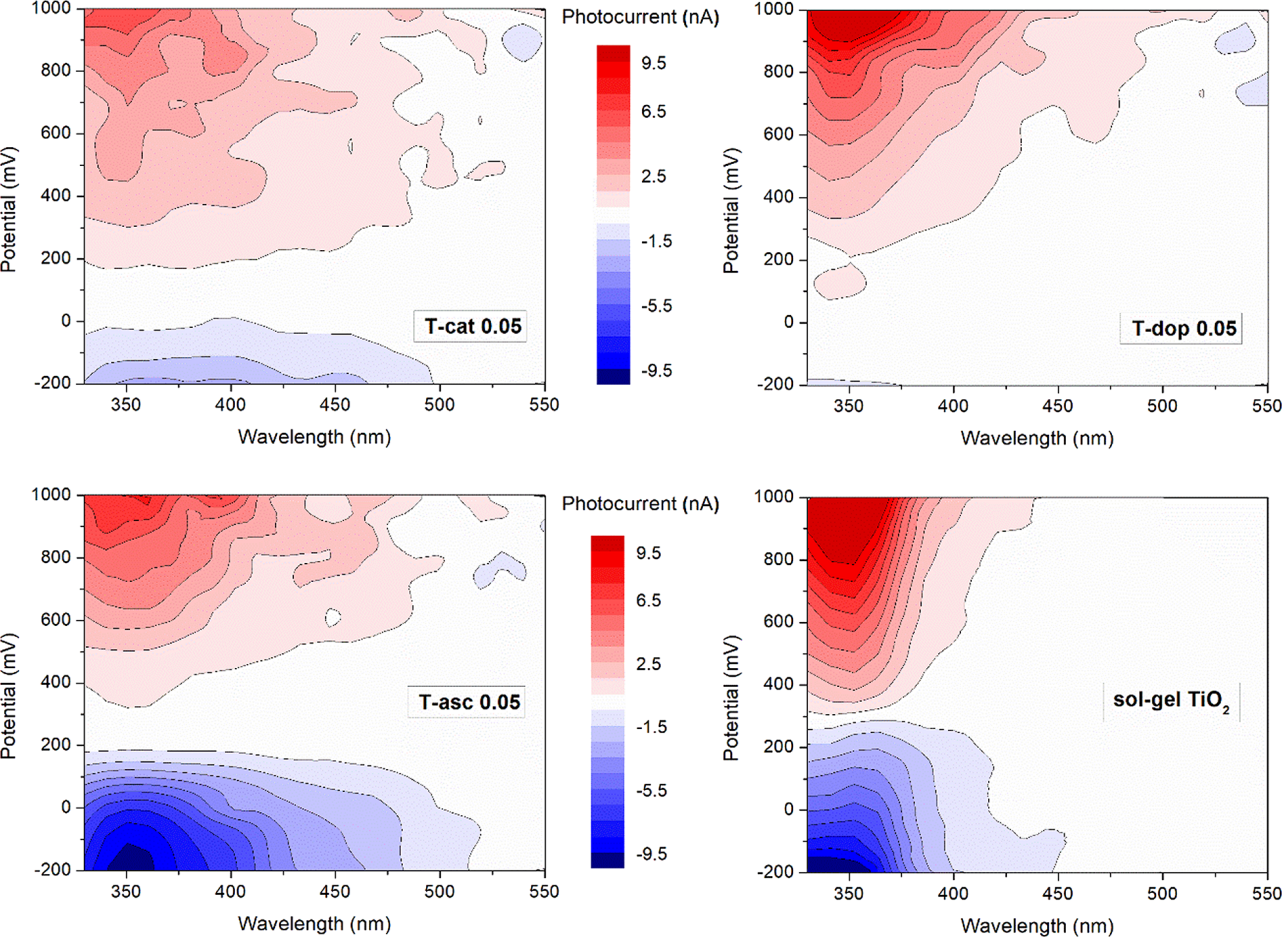
Photocurrent as a function of potential (vs Ag/AgCl) and incident light wavelength, recorded on PET/ITO electrodes covered with the hybrid samples (T-cat0.05, T-dop0.05, and T-asc0.05), in 0.1 M KNO_3_ aqueous solution electrolyte (pH 6.1), saturated with Ar.

The intense visible light absorption of the hybrid materials was correlated to their photo-response by photoelectrochemical measurements. The photocurrent data, collected as a function of the applied potential and irradiation wavelength in the same configuration used for CV measurements, are reported in Figure 6. In the three-dimensional maps, blue areas represent the cathodic photocurrent (corresponding to the reduction of an electron acceptor) and red areas represent the anodic photocurrent (oxidation of an electron donor, mainly water in aqueous electrolyte). For a better comparison of the photoactivity of the materials, representative action spectra showing the external quantum efficiency as a function of wavelength are reported in Figure S13. All the hybrid samples produce non-negligible photocurrents in the visible range, spread above 500 nm. This is a proof that the interfacial CT occurring in the xerogels allows an efficient separation of electron/hole pairs under visible light. It is worth noting that the detected photoactivity reaches about 520 nm, a narrower range compared to the band gap evaluated from the optical absorption edge. The weak photocurrents shown by the reference amorphous TiO_2_ above 400 nm may be related to band tails in its electron structure.

Different distributions of anodic and cathodic photocurrents are observed. T-dop0.05 shows almost exclusively anodic currents, reaching slightly higher values than T-cat0.05, while T-asc0.05 produces marked cathodic currents up to 200 mV, more intense than the anodic ones. A stronger oxidizing or reducing ability depends on the electronic structure of the semiconductor, on the band potentials and Fermi energy levels, as well as on its surface properties and modifications. In these experiments, argon was insufflated in the solution, evacuating O_2_, the major electron acceptor, so the cathodic photocurrent was supposed to be decreased. This points at a particularly high reducing efficiency of TiO_2_-*asc*.

In a photochemical redox process mediated by a hybrid semiconductor, the partially oxidized sensitizer may be reduced to its initial form by an electron transferred from the oxide or by a suitable electron donor present in the solution. Alternatively, further electron transfer steps might open self-degradation pathways, which depend on the functional groups and binding mode of the molecule^75^ and have been poorly investigated to date, although they represent a relevant issue for the stability of the sensitized oxide.

## 4 Conclusions

The one-pot sol–gel strategy described here is effective in the synthesis of amorphous titanium oxide with organic molecules coordinated to Ti ions. It allows a tunable functionalization and an accurate control of the organic content and product structure. For the three enediols considered (catechol, dopamine, and ascorbic acid), chemical, physical, and particulate gels can be produced by adjusting conditions such as the type of solvent and solution pH. The structure of the ligand affects the reactivity of the modified metal alkoxide and the evolution of the hybrid clusters composing the sol. In particular, the presence of additional functional groups on the ligand (as in dopamine and ascorbate) and its affinity with the solvent are crucial in modulating the rate of polycondensation and degree of cross-linking, aiding homogeneous gelation. These molecules can thus work as both stabilizing and functionalizing agents.

The stable ligand-to-metal charge transfer complexes induce effective photosensitization to the TiO_2_-based amorphous xerogels, as suggested by the intense absorption bands reaching 700 nm and attested by the generation of photocurrent under visible light irradiation. The ligands have different effects on the redox activity of the hybrid semiconductors: TiO_2_-dopamine and TiO_2_-ascorbate produce, respectively, stronger anodic (oxidizing) and cathodic (reducing) photocurrents than TiO_2_-catecholate. Room temperature EPR spectroscopy is a powerful tool to inspect the organic radicals formed by the interfacial charge separation and their evolution and stability in time. TiO_2_-catecholate samples are found to contain the most persistent radical species, whose concentration grows in time, an unexpected phenomenon likely related to the charge transfer equilibria and to reactions occurring between the ligands.

This kind of hybrid semiconductors may find application in photocatalysts, photoelectrodes, or sensors. Deeper EPR and photoelectrochemical studies could be synergically useful to further clarify the charge transfer processes and to monitor the behavior of such materials in operating conditions. Moreover, on the basis of the proposed synthetic approach, it is possible to prepare not only gels and particles but also stable sols for the deposition of hybrid coatings. Thus, it might be involved in the design of other metal oxide-organic systems in the form of nanostructured solids, powders, or films, with a uniformly distributed organic phase providing specific photoinduced and functional properties.

## Supporting Information Available

Synthesis conditions of all studied samples; photographs of wet and dried gels; and additional XRD, FTIR, TG-DTA, UV–vis DRS, EPR, and photocurrent data (PDF)
