# Supplementary material for: Interfacial Charge Transfer Complexes in TiO2-Enediol Hybrids Synthesized by Sol–Gel
Source: Langmuir. 2022 Jan 28;38(5):1821–32. doi: 10.1021/acs.langmuir.1c02939 (PMC8830207; doi:10.1021/acs.langmuir.1c02939)
Supplement: Supplementary file 1 — la1c02939_si_001.pdf [file la1c02939_si_001.pdf]

SUPPORTING INFORMATION

FOR

Interfacial Charge Transfer Complexes in TiO_2-Enediol Hybrids Synthesized by Sol-Gel

Claudio Imparato[a], Gerardino D'Errico[b], Wojciech Macyk[c], Marcin Kobielusz[c], Giuseppe Vitiello[a], and Antonio Aronne[a]*

[a] Department of Chemical, Materials and Production Engineering, University of Naples Federico II, P.le V. Tecchio 80, 80125 Napoli, Italy.

[b] Department of Chemical Sciences, University of Naples Federico II, Via Cinthia, 80126 Napoli, Italy.

[c] Faculty of Chemistry, Jagiellonian University, ul. Gronostajowa 2, 30-387 Kraków, Poland.

*Corresponding author. E-mail: claudio.imparato@unina.it

Table S1. Synthesis conditions of all the hybrid TiO_2 samples prepared with catecholate (cat), dopamine anion (dop) or ascorbate (asc) as ligands.

**Sample**	***c* = ligand/Ti** (mol/mol)	**[Ti]** (mol/L)	***h* = H_2O/Ti** (mol/mol)	**solvent*, additives**	**product**	**gelation time**
**T-cat0.01p**	0.01	0.85	4		precipitate	-
**T-cat0.05p**	0.05	0.85	4		precipitate	-
**T-cat0.1p**	0.10	0.85	4		precipitate	-
**T-cat0.2p**	0.20	0.85	4		physical gel	-
**T-cat0.4p**	0.40	0.85	4		physical gel	-
**T-cat0.05**	0.05	0.57	2	1-propanol/ cyclohexane, HCl	chemical gel	1 h
**T-cat0.1**	0.10	0.57	2	1-propanol/ cyclohexane, HCl	chemical gel	1 day
**T-cat0.1A**	0.10	0.85	4	*Hacac*, HCl, NH_3	chemical gel	5 min
**T-cat0.1C**	0.10	0.52	2	citric acid	chemical gel	10 min
**T-cat0.1D**	0.10	0.52	2	diethanolamine	chemical gel	10 min
**T-dop0.05p**	0.05	0.52	4	NH_3	precipitate	-
**T-dop0.05**	0.05	0.52	4		chemical gel	15 min
**T-dop0.1**	0.10	0.38	4		chemical gel	2 days
**T-asc0.1p**	0.10	0.30	4	ethanol, HCl	precipitate	-
**T-asc0.05**	0.05	0.30	4	HCl	chemical gel	7 days
**T-asc0.1**	0.10	0.30	4	HCl	chemical gel	3 days
**T-asc0.2**	0.20	0.30	4	HCl	chemical gel	3 h

* unless otherwise noted, the solvent is 1-propanol.

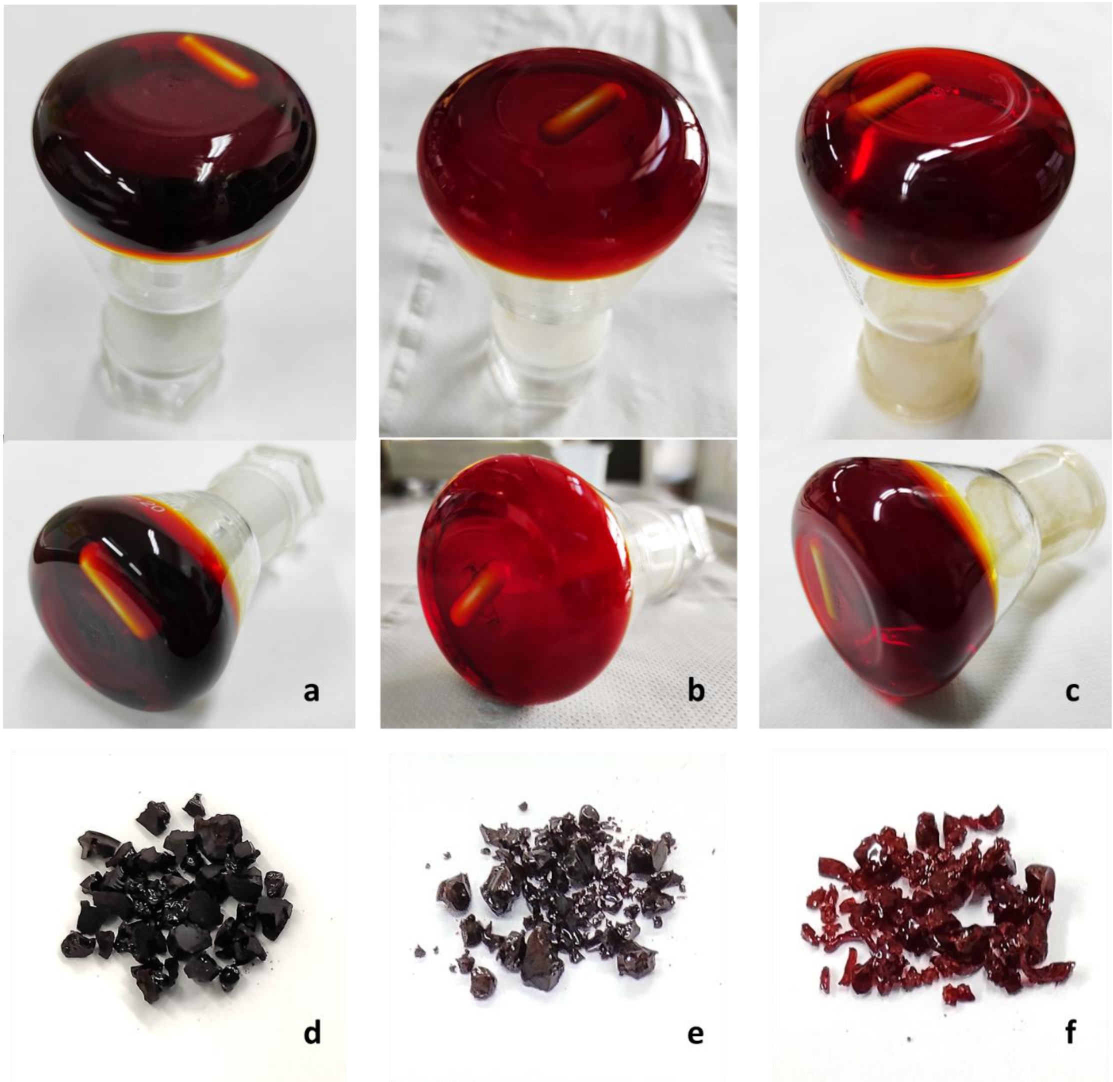

Figure S1. Photographs of hybrid wet gels: T-cat0.1 (a), T-dop0.1 (b) and T-asc0.1 (c) and of the corresponding xerogels: T-cat0.1 (d), T-dop0.1 (e) and T-asc0.1 (f).

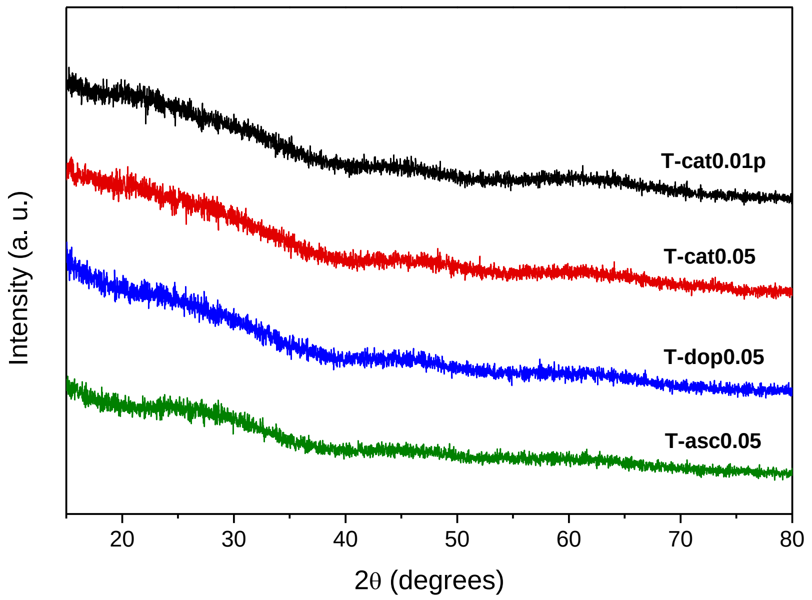

Figure S2. X-ray diffraction (XRD) profiles of representative hybrid TiO_2 samples.

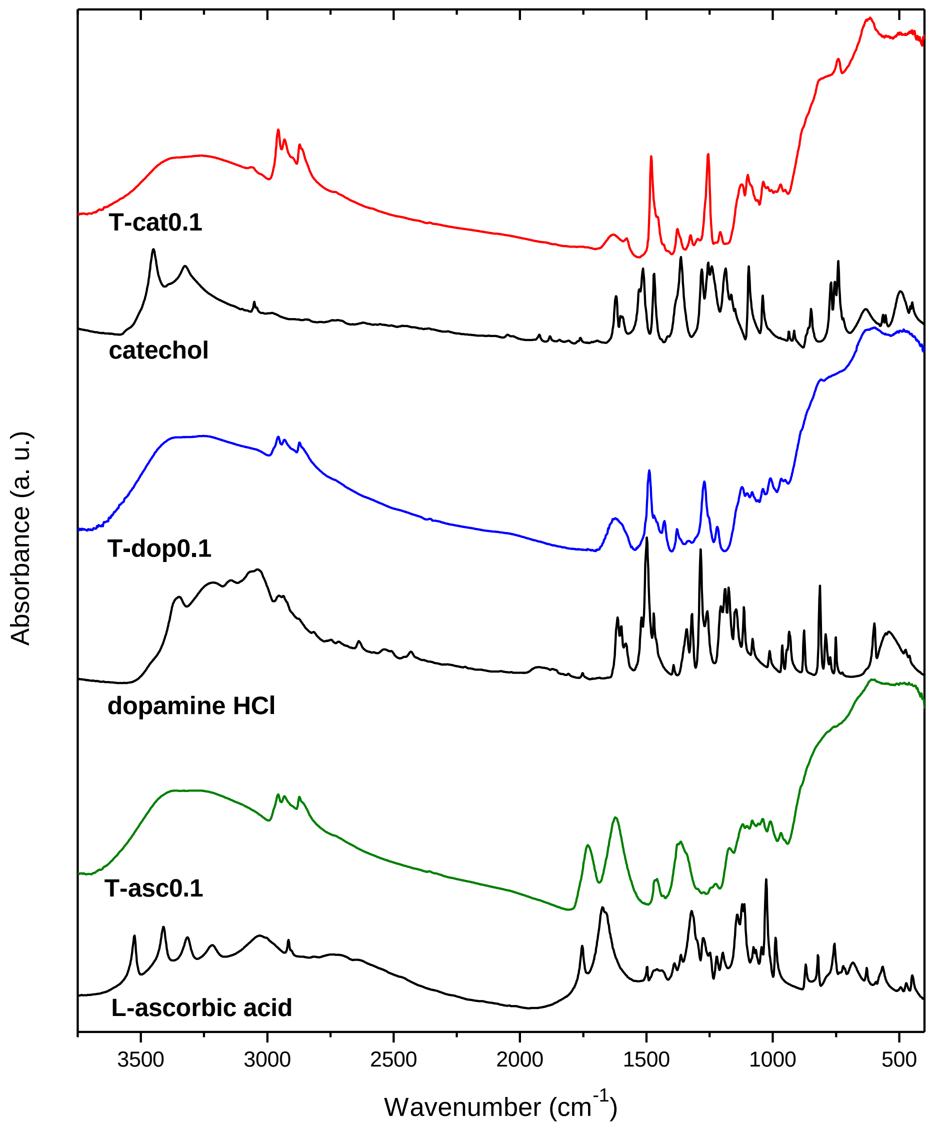

Figure S3. FTIR spectra of representative hybrid xerogels and of the pure organic molecules used as ligands.

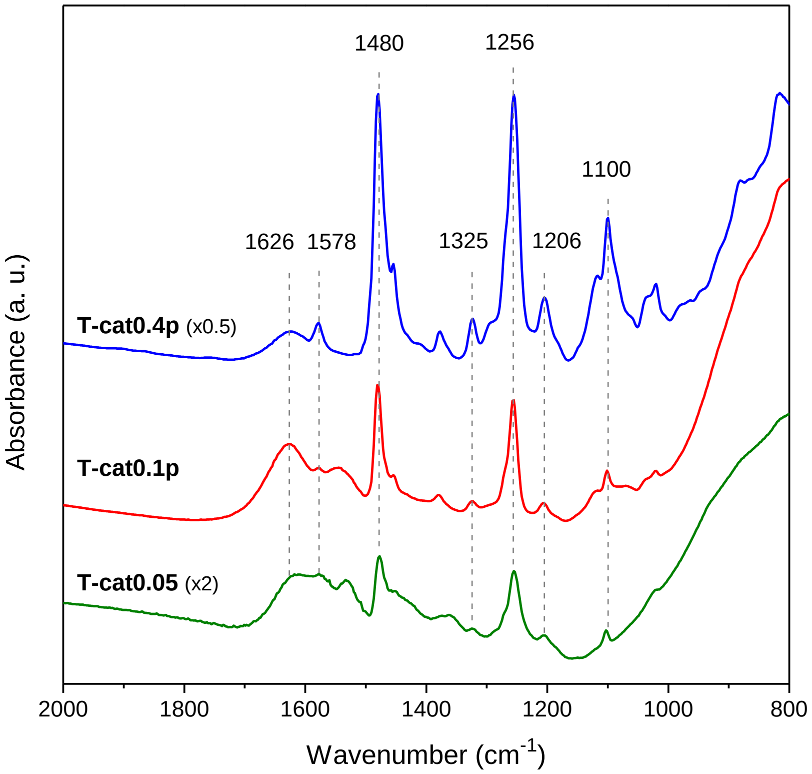

Figure S4. FTIR spectra of TiO_2-catecholate samples synthesized with different catechol/Ti molar ratios.

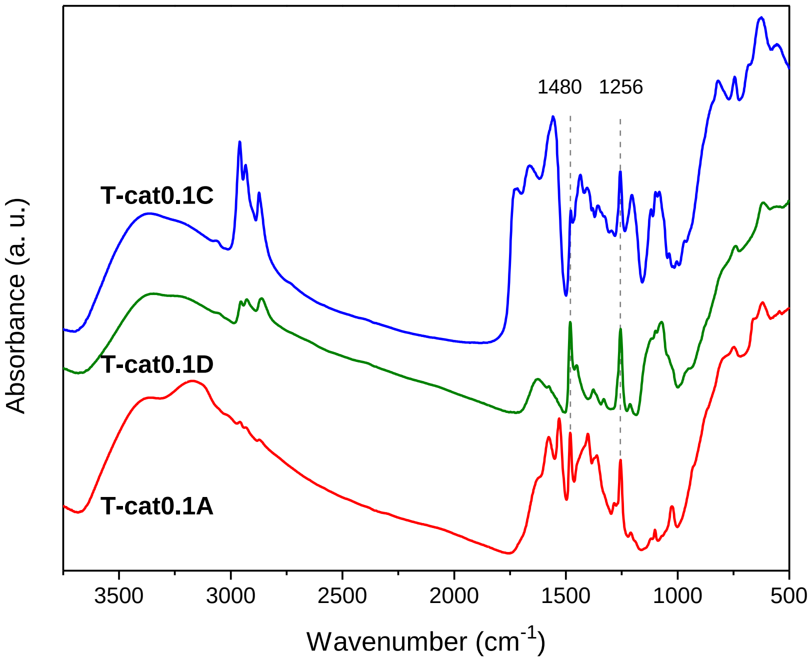

Figure S5. FTIR spectra of TiO_2-catecholate mixed xerogels containing acetylacetone (T-cat0.1A), diethanolamine (T-cat0.1D) or citric acid (T-cat0.1C) as additional ligand.

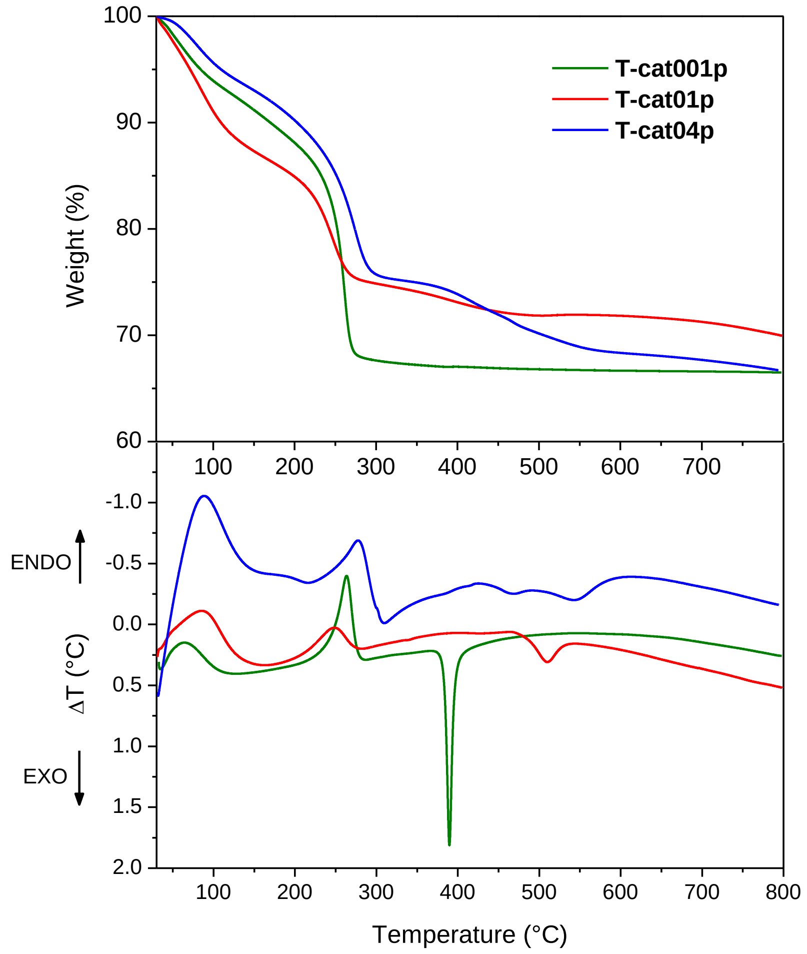

Figure S6. TGA (top) and DTA (bottom) profiles of TiO_2-catecholate samples synthesized with different catechol/Ti molar ratios, recorded in N_2 at 10 °C/min heating rate

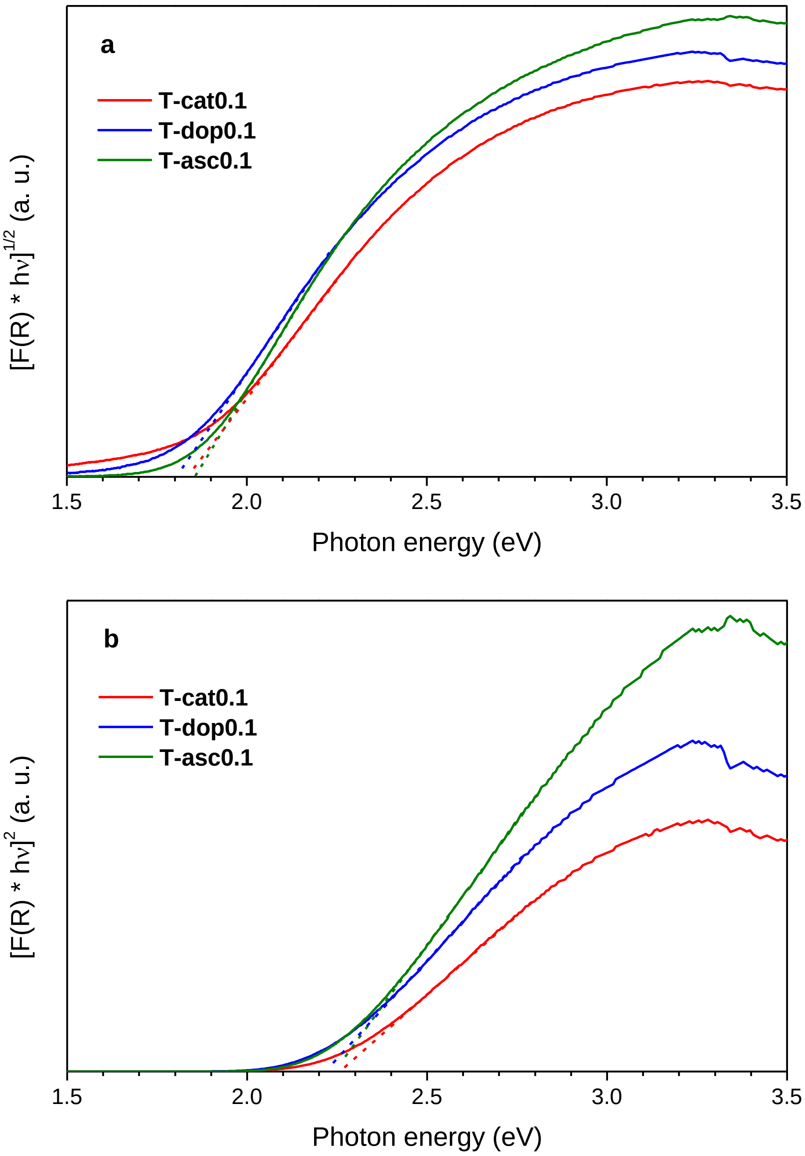

Figure S7. Tauc plots derived from UV-vis diffuse reflectance spectra for indirect (a) and direct (b) band gap.

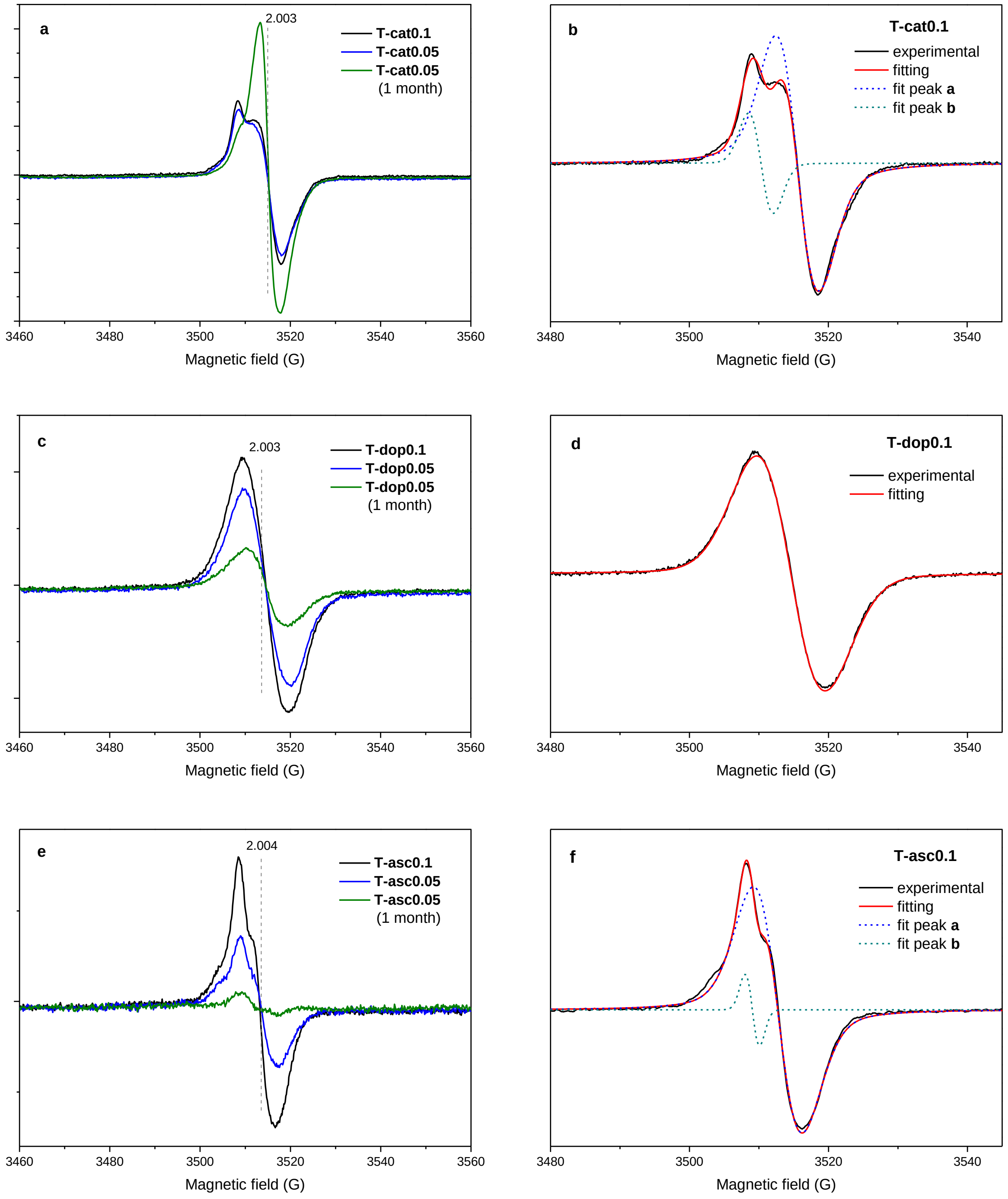

Figure S8. EPR spectra recorded at room temperature on TiO_2-cat (a), TiO_2-dop (c) and TiO_2-asc (e) samples prepared with ligand/Ti molar ratios equal to 0.10 and 0.05, and on the latter after 1 month storage; results of 2-peaks line fitting for T-cat0.1 (b) and T-asc0.1 (f) and single peak fitting for T-dop0.1 (d).

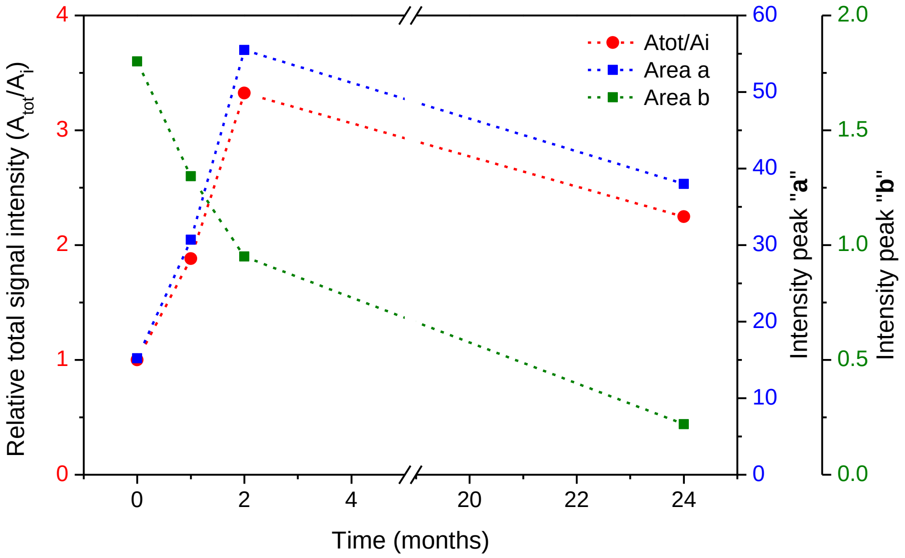

Figure S9. Analysis of the EPR signal of T-cat0.01p sample in time: total intensity relative to the initial total area obtained by double integration of the spectrum and intensity of the “a” and “b” components evaluated by line fitting.

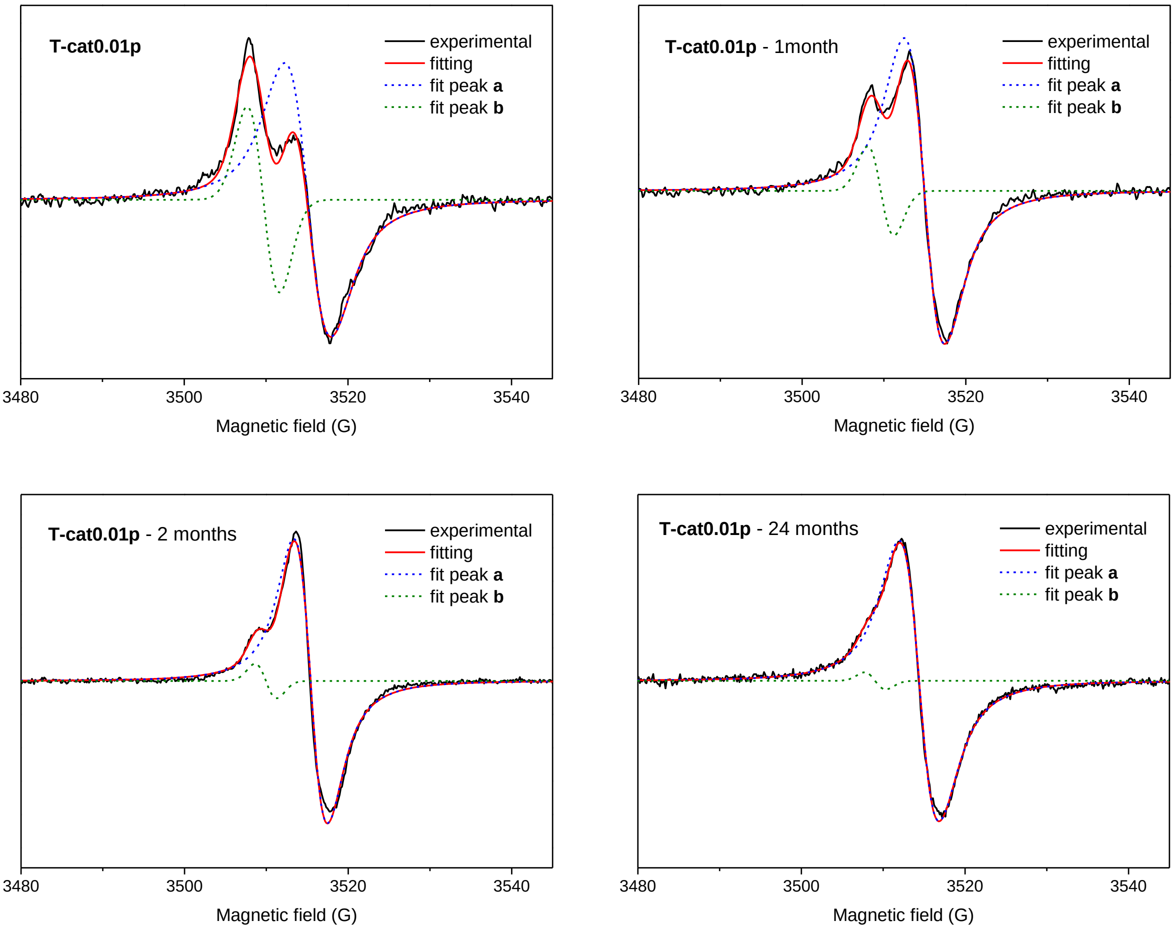

Figure S10. EPR spectra recorded at room temperature on T-cat0.01p sample as prepared and after storage for different times in ambient conditions, with the corresponding 2-peaks line fitting.

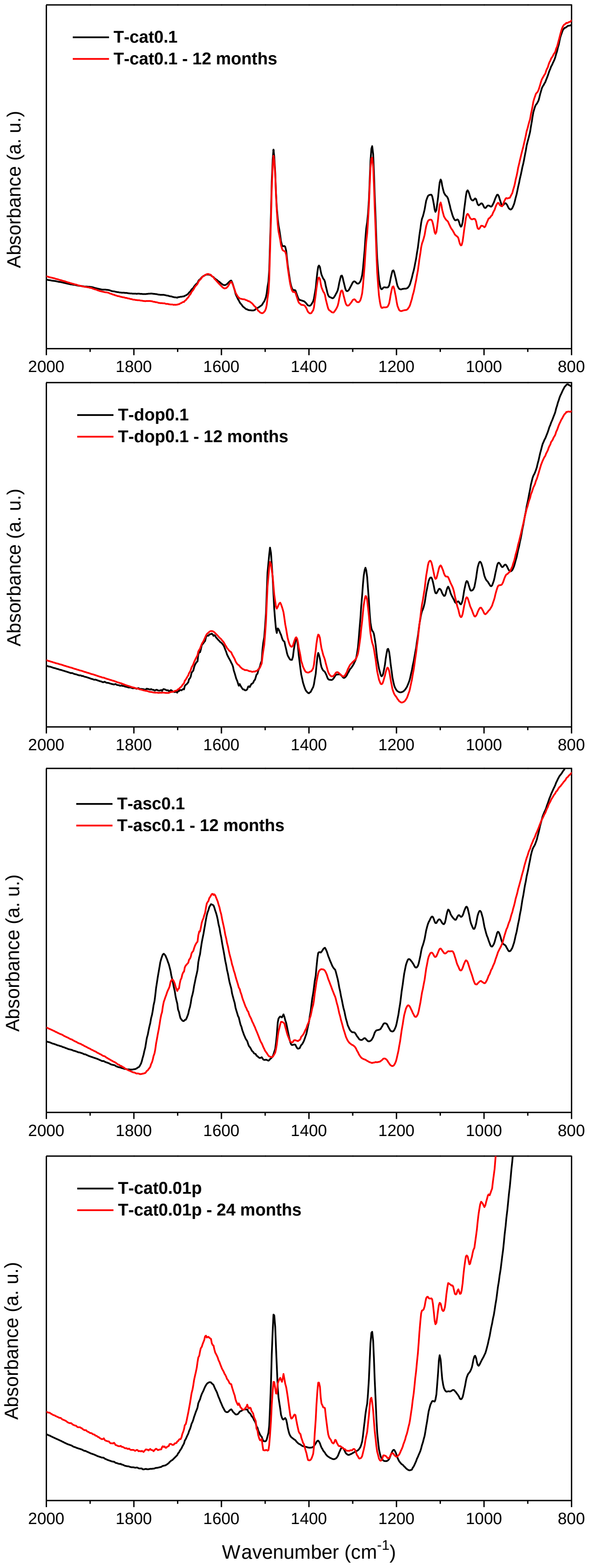

Figure S11. FTIR spectra of hybrid samples as prepared and after storage for 12 or 24 months.

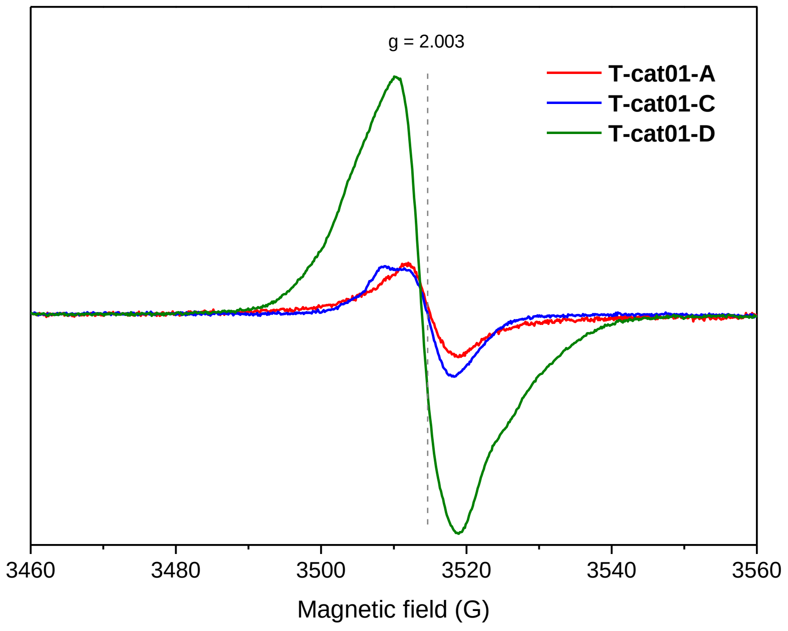

Figure S12. EPR spectra recorded at room temperature on TiO_2-catecholate mixed xerogels containing acetylacetone (T-cat0.1A), diethanolamine (T-cat0.1D) or citric acid (T-cat0.1C) as additional ligand.

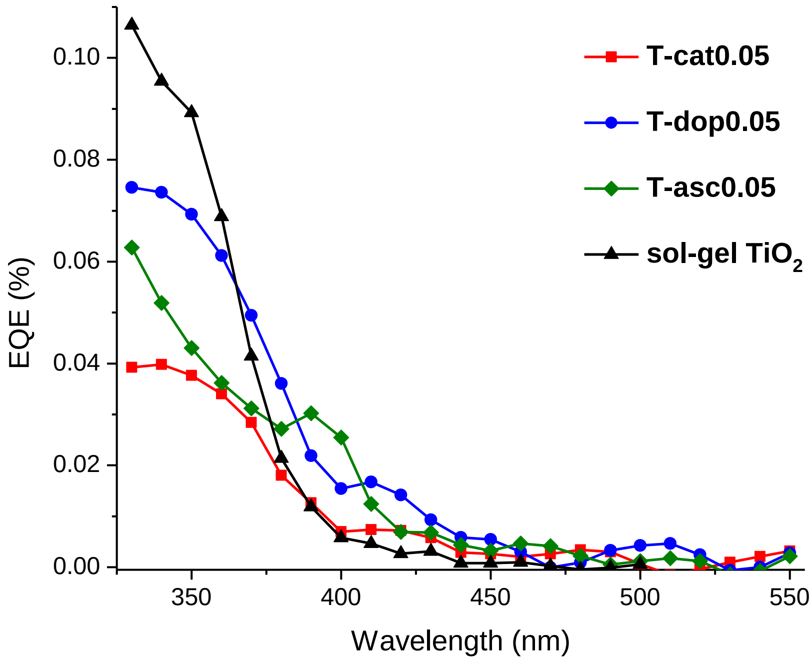

Figure S13. The external quantum efficiency (EQE) as function of wavelength for the studied materials at 1.0 V vs. Ag/AgCl. Recorded on PET/ITO electrodes coated with the hybrid samples (T-cat0.05, T-dop0.05, T-asc0.05) and reference TiO_2, in 0.1 M KNO_3 aqueous solution electrolyte (pH = 6.1), saturated with Ar.
